# Molecular Insights into the Assembly and Functional Diversification of Typhoid Toxin

**DOI:** 10.1128/mbio.01916-21

**Published:** 2022-01-11

**Authors:** Xiaoyu Liu, Zhe Chen, Xuyao Jiao, Xukai Jiang, Jicheng Qiu, Fuping You, Hongan Long, Hongzhi Cao, Casey C. Fowler, Xiang Gao

**Affiliations:** a State Key Laboratory of Microbial Technology, Shandong University, Qingdao, China; b National Glycoengineering Research Center, Shandong University, Qingdao, China; c School of Life Sciences, Shandong University, Qingdao, China; d Institution of System Biomedicine, Peking University, Beijing, China; e Institute of Evolution and Marine Biodiversity, KLMME, Ocean University of China, Qingdao, China; f Department of Biological Sciences, University of Alberta, Edmonton, Alberta, Canada; Duke University School of Medicine

**Keywords:** *Salmonella* Typhi, typhoid fever, bacterial pathogenesis, bacterial toxins, glycobiology, AB_5_ toxins

## Abstract

Typhoid toxin is an A_2_B_5_ protein toxin and an important virulence factor for the human-adapted bacterial pathogen Salmonella enterica serovar Typhi, the causative agent of typhoid fever. Typhoid toxin contains two enzymatic subunits, PltA and CdtB, which dock onto a pentameric delivery platform composed of the protein PltB. It was recently reported that the same enzymatic subunits can assemble with a different delivery platform composed of the protein PltC, forming a distinct version of typhoid toxin. However, the differences in structure and receptor specificity between the PltC and PltB typhoid toxins remain unknown. Here, we determined atomic-level structures of the pentameric PltC subunit, the fully assembled PltC typhoid toxin, and the PltC pentamers in complex with glycan receptors. Biochemical and structural analyses indicate that PltB and PltC are unable to form heteromeric delivery complexes due to electrostatic repulsion at the subunit interface and thus form separate toxins only. We further observed that, despite low sequence similarity between PltB and PltC, they interact with PltA in a similar manner but that PltC exhibits stronger electrostatic interactions with PltA, enabling it to outcompete PltB in toxin assembly. The ligand-bound atomic structures of PltC show an additional glycan binding site not found in PltB and glycan array analysis indicates that PltB and PltC exhibit significant differences in glycan binding specificity. Collectively, this study offers atomic-level insights into how *S*. Typhi produces two distinct versions of typhoid toxin, thereby generating functional diversity in this key virulence factor.

## INTRODUCTION

Salmonella enterica serovar Typhi is an exclusively human pathogen and the etiological agent of typhoid fever, a significant global public health concern, which causes around 11 million human infections and results in more than 115,000 deaths worldwide every year (1–3). Upon invasion of human cells, *S*. Typhi produces and secretes high levels of a unique AB-type protein toxin, typhoid toxin, which induces widespread cell cycle arrest, and in some cases apoptosis, in intoxicated host cells (4–9). Cell culture and animal models of infection and intoxication indicate that typhoid toxin is a central virulence factor for *S*. Typhi that may contribute directly to the symptomology of typhoid fever (9–11). Typhoid toxin is a member of the AB_5_ family of bacterial toxins, which also includes other medically relevant toxins, such as pertussis toxin, Shiga toxin, and cholera toxin (12–17). This class of toxins is defined by the common structure adopted by this evolutionarily diverse class of toxins that features a single A (active) subunit associated with a pentameric receptor-binding B (delivery) subunit (15). However, unlike other members of this family, typhoid toxin displays an unprecedented A_2_B_5_ architecture in which two active subunits (CdtB and PltA) are associated with the pentameric delivery complex (9). CdtB, a homolog of the active subunit of cytolethal distending toxin (CDT), is a DNase that introduces double-stranded breaks into host cell DNA, causing cell cycle arrest and cellular distension (18, 19). PltA is an ADP ribosyltransferase; however, its target(s) and biological function have not yet been identified (8). CdtB and PltA are covalently linked via a disulfide bond; the evolution of this bond is proposed to be a key step in the emergence of typhoid toxin from an AB_5_ toxin ancestor (10).

Typhoid toxin was originally identified and characterized as having a homopentameric delivery platform composed of PltB (8). As in other AB_5_-type toxins, PltB associates with its A subunit noncovalently via the insertion of a C-terminal α-helix of PltA into the pore at the center of the PltB pentamer (9, 15). PltB has a canonical glycan binding pocket in each monomer that specifically binds *N*-acetylneuraminic acid (Neu5Ac)-terminated glycans; the interaction of PltB with Neu5Ac-decorated glycans on the surface of human cells mediates the delivery of this toxin during *S*. Typhi infection (9, 20–22). The receptor binding specificity of the B subunit therefore defines the *in vivo* tropism of the toxin by dictating the cell and tissue types that are intoxicated and is also central to the host adaptation of typhoid toxin (10, 11, 20, 23, 24). Indeed, a recent study found that typhoid toxin encoded by Salmonella enterica serovar Javiana, which shares 99% sequence similarity to *S*. Typhi typhoid toxin, fails to produce the characteristic manifestations of typhoid fever elicited by *S*. Typhi typhoid toxin in mouse models due to subtle changes in the PltB sequence that confer distinct glycan-binding specificities (25).

Recently, it was discovered that, in addition to the originally identified (PltB) version of typhoid toxin, *S*. Typhi also produces a second typhoid toxin composed of the same A subunits (PltA and CdtB) in complex with a distinct delivery subunit, a previously uncharacterized protein that has been named PltC (23). A similar situation has been reported in *S*. Javiana (26, 27). *pltC* is found at a genomic locus that is distant from all other typhoid toxin-related genes, where it resides immediately downstream of an ADP-ribosyltransferase pseudogene, *sty1362* (23). The *pltC*/*sty1362* locus is homologous to the *artA*/*artB* locus, which encodes the ArtAB toxin, a distinct AB_5_-type toxin produced by a range of *Enterobacteriaceae* (28–30). PltC therefore likely emerged within a strain encoding both typhoid toxin and ArtAB wherein the ArtB subunit was repurposed to act as an alternate delivery platform for typhoid toxin and the *artA* gene was degraded. Interestingly, our previous study showed that ArtB from Salmonella enterica serovar Typhimurium DT104 could also form a functional chimeric typhoid toxin with PltA-CdtB from *S*. Typhi (10).

Typhoid toxin containing PltC has properties that are significantly different from those of the PltB version of the toxin (23). Using genetic deletions and purified recombinant toxins, it was shown that PltC typhoid toxin was less potent at inducing cell cycle arrest in a human epithelial cell line than the PltB toxin. Animal models show a very different response to PltC and PltB toxin administration: the PltC toxin is more potent at reducing the numbers of circulating immune cells but elicited reduced overt symptomology and reduced mortality compared to the PltB toxin at the doses tested. Intriguingly, there was also a notable difference in the trafficking of the two toxins during infection. Typhoid toxin is produced by *S*. Typhi organisms that have invaded a human cell and reside within an intracellular Salmonella-containing vacuole (SCV); while the PltB version of typhoid toxin engages glycosylated host receptors within the SCV to trigger its exocytosis from the cell, the PltC version of the toxin remains associated with the SCV in a human epithelial cell model of infection. The assembly of different toxins therefore appears to confer functional versatility to typhoid toxin that likely plays an important role in *S*. Typhi virulence. However, the molecular mechanisms of assembling different typhoid toxins and the differences in glycan-binding properties that presumably drive the observed functional versatility are currently unknown.

Here, we present the structures of the PltC homopentamer, the PltC typhoid toxin, and the glycan-bound PltC delivery platform. We utilized biochemical assays to analyze the interactions of the typhoid toxin B subunits with each other, with their A subunits, and with diverse glycans. Collectively, our data provide an atomic-level view and a mechanistic understanding of how *S*. Typhi produces two functionally versatile typhoid toxins.

## RESULTS

### PltC and PltB form homopentameric delivery platforms and distinct typhoid toxins.

**FIG 1:**
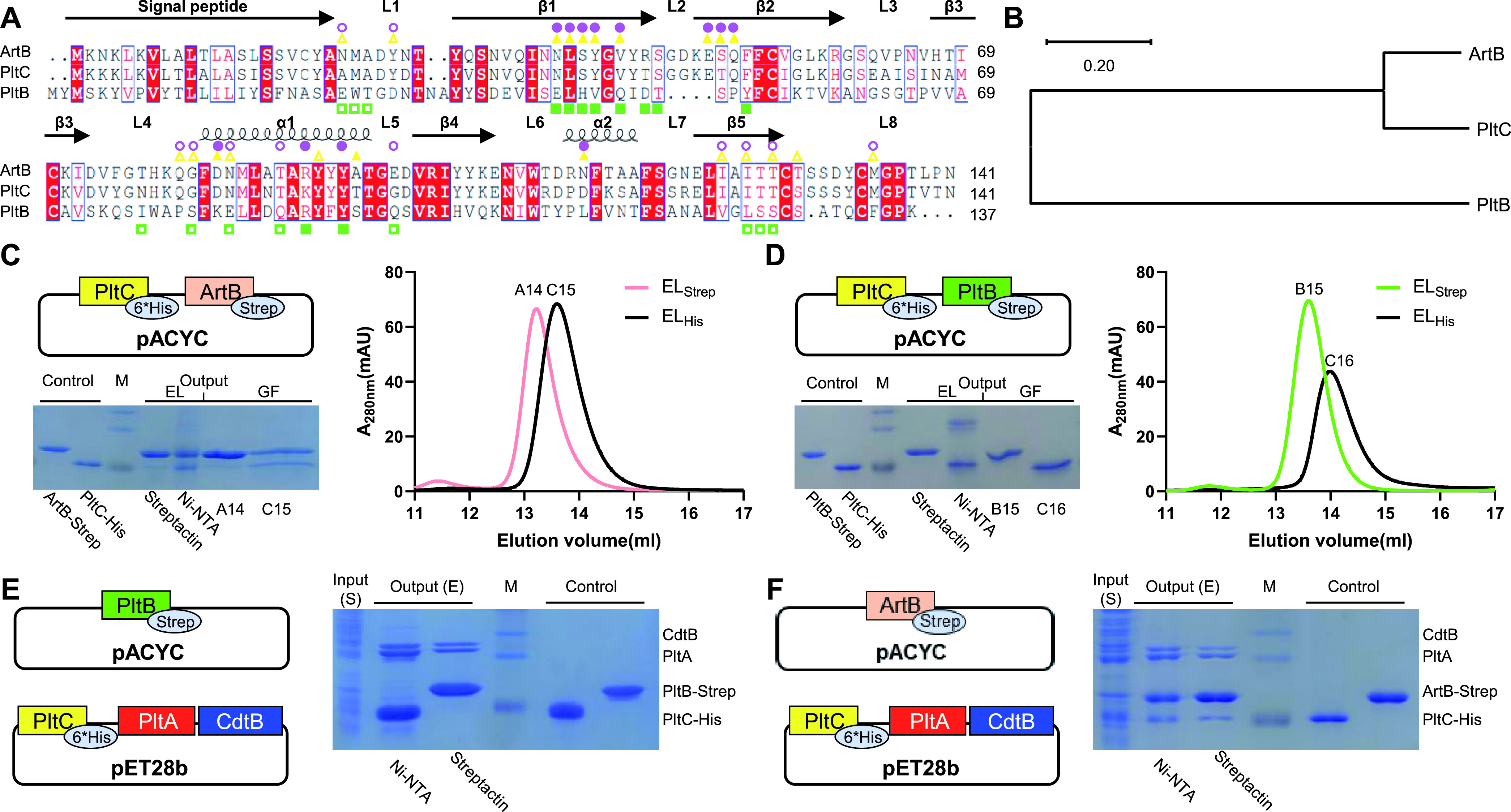
PltB and PltC form distinct homopentameric delivery platforms for typhoid toxin. (A) Protein sequence alignment of PltB, PltC (both from *S*. Typhi CT18), and ArtB (*S.* Typhimurium DT104). Identical and similar residues among all three proteins are indicated by white text with a red background and red text, respectively. Secondary structures were assigned based on the structure of PltB. The amino acid residues involved in the subunit interface are highlighted. Green squares, PltB; yellow triangles, PltC; purple circles, ArtB. Empty and filled shapes indicate the amino acid residues from two adjacent interaction subunits. (B) Phylogenetic analysis of PltB, PltC, and ArtB using the maximum-likelihood method conducted in MEGA X. (C) Coexpressed PltC and ArtB form heteropentamers. PltC-His_6_ and ArtB-Strep were coexpressed from the pACYCDuet vector. Bacterial cell lysate was divided equally into two portions and loaded onto columns filled with Ni-NTA resin or Strep-Tactin resin. The elution samples (EL) from affinity purification were analyzed by SDS-PAGE and were further monitored by gel filtration (GF) to analyze the composition of the pentamer. The peak fractions of two EL samples from GF were also analyzed by SDS-PAGE. (D) Coexpressed PltC and PltB form distinct homopentamers. The experiment described for panel C was repeated using PltB-Strep in place of ArtB-Strep. (E) Coexpressed PltC and PltB form distinct homopentamers only in the presence of PltA-CdtB. PltC-His_6_-PltA-CdtB and PltB-Strep were coexpressed with pET28b and pACYCDuet vectors, respectively. Soluble cell lysate (S) was divided equally into two portions and loaded onto columns filled with Ni-NTA resin or Strep-Tactin resin. The elution samples (E) from affinity purification were analyzed by SDS-PAGE. (F) Coexpressed PltC and ArtB could form heteropentamers in the presence of PltA-CdtB. The experiment described for panel E was repeated using ArtB-Strep in place of PltB-Strep. For panels C to F, control samples were purified PltC-His_6_, PltB-Strep, or ArtB-Strep, as indicated; M, molecular weight marker. All experiments were carried out at least three times with consistent results.

The presence of two different delivery subunits for typhoid toxin evokes important questions about the mechanisms of toxin assembly, most notably whether the different B subunits form distinct homopentameric delivery platforms, heteropentameric platforms, or both. Previous work demonstrated that *S*. Typhi can produce typhoid toxins containing homopentameric delivery platforms composed of PltB and PltC during infection and failed to detect an interaction between PltB and PltC, suggesting that toxins containing heteromeric delivery complexes do not form or are very scarce under the conditions tested (23). A recent study looking at the interaction of the PltB and PltC homologs found in *S*. Javiana within a heterologous host system concluded that PltB and PltC might be able to form heteromeric toxins, although the heteromeric B subunit interactions were observed to be notably weaker than those of the homomeric interactions (26). In order to further explore the molecular mechanisms of B subunit assembly in typhoid toxin, we developed an affinity purification-based interaction assay wherein different B subunits tagged with 6×His and streptavidin (Strep) affinity tags, respectively, were coexpressed and affinity purified in a heterologous Escherichia coli host system. To evaluate our assay, we first tested to see whether PltC could form heteromeric delivery platforms with its close evolutionary cousin ArtB. Although PltB and PltC are encoded by the same strain, amino acid sequence alignment and phylogenetic analysis of PltB, PltC, and ArtB show that PltC and ArtB share much higher sequence similarity and have a closer evolutionary relationship and that PltB was evolutionarily segregated with the common ancestor of PltC and ArtB (Fig. 1A and B). We coexpressed PltC-His_6_ and ArtB-Strep, applied an equal amount of clarified bacterial lysate to nickel-nitrilotriacetic acid (Ni-NTA) and Strep-Tactin resins, performed affinity purification and then analyzed elution samples from both resins using SDS-PAGE. We found that both the Ni-NTA and the Strep-Tactin preparations contained a mixture of PltC-His_6_ and ArtB-Strep, indicating that the two B subunits copurify and suggesting that they are able to form a complex (Fig. 1C). To further explore the protein behavior and oligomeric state of these proteins, we analyzed the elution samples using size exclusion chromatography, and the results indicated that PltC and ArtB form heteropentamers (Fig. 1C). Notably, the relative compositions of PltC and ArtB present differently in both elution samples and gel filtration samples on SDS-PAGE depending on the types of the affinity purification we used, which implies that PltC and ArtB may form different heteropentamers in diverse ratios (Fig. 1C). We then conducted a similar experiment using PltC-His_6_ and PltB-Strep to determine whether PltB and PltC can also form heteromeric complexes. However, we observed that the Ni-NTA preparation contained only PltC-His_6_ and the Strep-Tactin preparation contained only PltB-Strep, indicating that PltC and PltB do not interact. Size exclusion chromatography indicated that, as expected, PltB and PltC both form stable homogeneous pentamers (Fig. 1D). To determine whether the A subunits impact the composition and assembly of the B subunits, we coexpressed PltA-CdtB with PltC-His_6_ and PltB-Strep or ArtB-Strep and reproduced the affinity purification with the different resins as described above. As expected, CdtB-PltA forms an A_2_B_5_ typhoid toxin with either a PltB homopentamer or a PltC homopentamer, but we were unable to detect toxin containing PltB/PltC heteropentameric delivery platforms (Fig. 1E). However, consistent with the observations above, we found that ArtB and PltC form a heteropentamer with CdtB-PltA incorporated (Fig. 1F). Collectively, these results are congruent with previous results that indicate that *S*. Typhi produces PltC and PltB versions of typhoid toxin but that PltC and PltB are unable to assemble into heteromeric delivery complexes.

### Electrostatic repulsion prevents the formation of typhoid toxins containing heteromeric delivery platforms.

**FIG 2:**
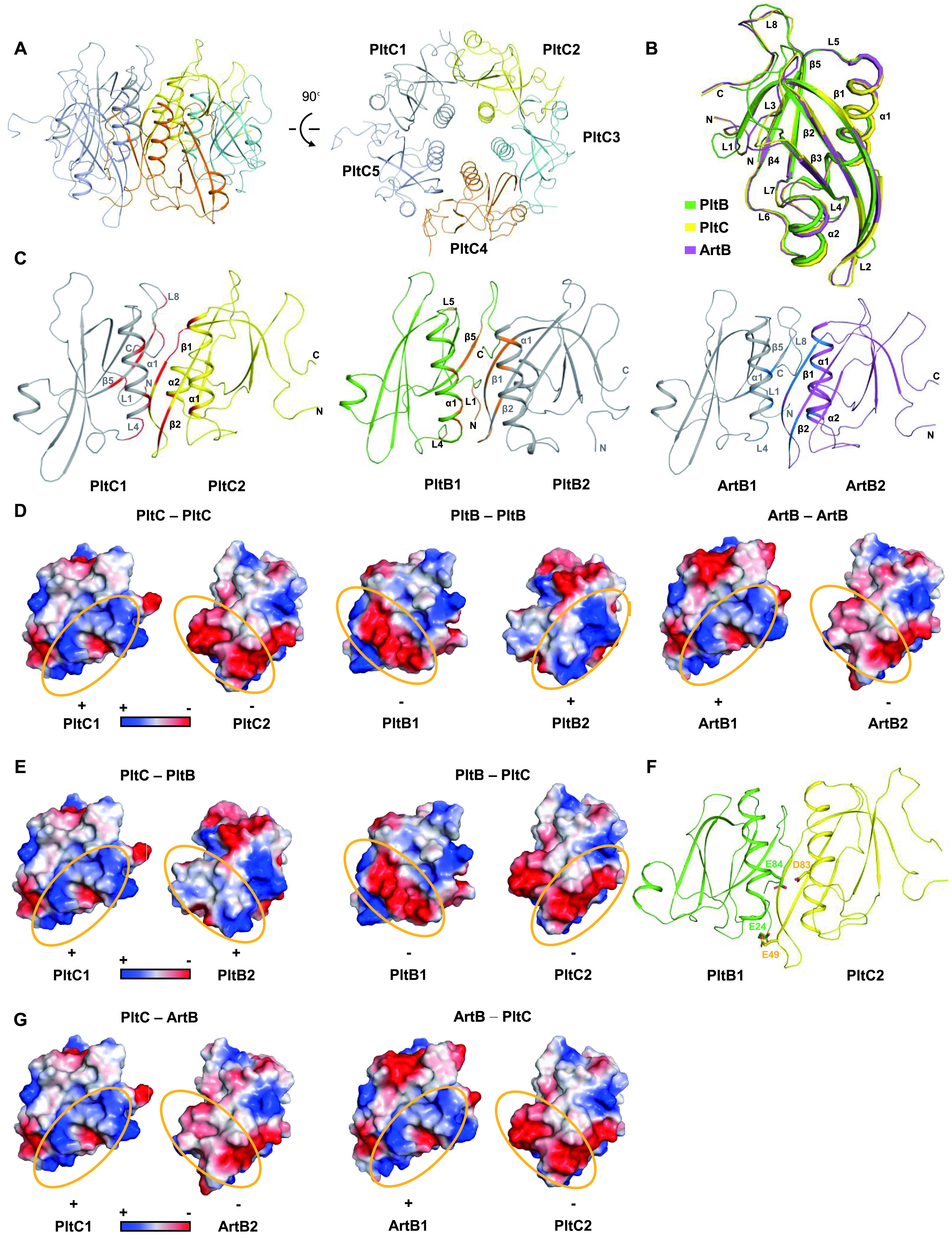
Electrostatic interactions at the subunit interface impede the formation of PltC-PltB heteropentamers. (A) Two views of the atomic structure of PltC homopentamer shown as a ribbon cartoon and representing a 90° rotation about a horizontal axis. Each monomer is displayed using a different color. (B) Structural alignment between PltC, PltB, and ArtB shown using a monomer. (C) Oligomerization interfaces of PltC, PltB, and ArtB are shown as ribbon cartoons and indicated with different colors. Secondary structures involved in the intersubunit interactions are labeled. (D) Surface charge distribution of the interaction surfaces of two adjacent monomers of PltC, PltB, and ArtB. The areas enclosed by yellow ellipses show a key interface where attraction between positive (blue) and negative (red) potentials underlies key electrostatic interactions between adjacent subunits. (E and F) A PltC-PltB heterodimer modeled in PyMOL. (E) Surface charge distribution of the interaction surfaces of PltC-PltB. The area enclosed by the yellow ellipses shows a mutual repulsion potential between the two monomers. (F) Cartoon of the PltB-PltC heterodimer highlighting two pairs of amino acid residues (as sticks) that exhibit electrostatic repulsion in this model. (G) A PltC-ArtB heterodimer modeled in PyMOL. The surface charge distribution of the interaction surfaces of PltC-ArtB shows attraction between positive and negative potentials of the two monomers.

To gain insight into the molecular mechanism of the pentamerization of B subunits, we determined the crystal structure of the PltC homopentamer at a resolution of 1.33 Å (Fig. 2A and Table S1) and compared this to analogous structures of PltB and ArtB homopentamers solved previously (10, 20). Structural comparison of the monomer structures of PltB, PltC, and ArtB indicate that, while all three proteins adopt a similar overall structure, PltC shares a higher structural similarity to ArtB with a root mean square deviation (RMSD) of 0.213 Å over 100 Cα atoms compared to PltB, with a RMSD of 0.642 Å over 88 Cα atoms (Fig. 2B), which is consistent with the fact that sequence identity between PltC and ArtB (∼73%) is much higher than that between PltC and PltB (∼31%) (Fig. 1A). These large structural and sequence differences between PltB and PltC likely contribute to the inability of PltB and PltC to form a heterocomplex. Close inspection of their oligomerization interfaces revealed that the monomers of PltC, PltB, and ArtB interact via similar structural elements—mainly loops L1 and L2, β strands β1, β2, and β5 and helix α1—to form a homopentamer (Fig. 2C and Fig. 1A). However, while there is appreciable overlap in the intersubunit interactions of ArtB and PltC, the specific residues and chemical interactions involved in the intersubunit interactions of the PltB homopentamer differ substantially (Fig. 1A and Fig. S1 to S3). Electrostatic surface potential analysis of the interfaces of PltC-PltC, PltB-PltB, and ArtB-ArtB shows that it is mainly electrostatic interactions that facilitate subunit pentamerization (Fig. 2D). Importantly, the charge distribution of the PltB interface is substantially different from that of the PltC or ArtB interface. We therefore modeled PltC-PltB and PltC-ArtB heteromeric complexes using PyMOL to explore their subunit interfaces. Consistent with our observations above that PltB and PltC do not interact, the interfaces between PltC and PltB exhibit significant electrostatic repulsion, most notably via D83_PltC_-E84_PltB_ and E49_PltC_-E24_PltB_ (Fig. 2E and F), which likely presents a substantial barrier to PltC-PltB complex formation. In contrast, the PltC-ArtB interface shows electrostatic attraction interactions, consistent with our observations that these proteins can form heteropentamers (Fig. 2G). Together, these results indicate that *S*. Typhi produces distinct PltB and PltC versions of typhoid toxin and that electrostatic repulsion at the subunit interface likely plays a major role in preventing the formation of heteromeric delivery platforms.

10.1128/mBio.01916-21.1 FIG S1 Interacting amino acids at the interface of the PltC homodimer. The insets of the blue box (A) and light blue box (B) represent the interacting amino acids in the upper part and lower part of the PltC dimer, respectively. Interacting residues are shown as sticks; interactions are shown as black dashes. Download FIG S1, PDF file, 0.2 MB. Copyright © 2022 Liu et al. 2022 Liu et al. https://creativecommons.org/licenses/by/4.0/ This content is distributed under the terms of the Creative Commons Attribution 4.0 International license.

10.1128/mBio.01916-21.9 TABLE S1 Data collection and refinement statistics. Download Table S1, DOCX file, 0.02 MB. Copyright © 2022 Liu et al. 2022 Liu et al. https://creativecommons.org/licenses/by/4.0/ This content is distributed under the terms of the Creative Commons Attribution 4.0 International license.

### The global structure of the PltC typhoid toxin exhibits striking similarity to the PltB typhoid toxin.

**FIG 3:**
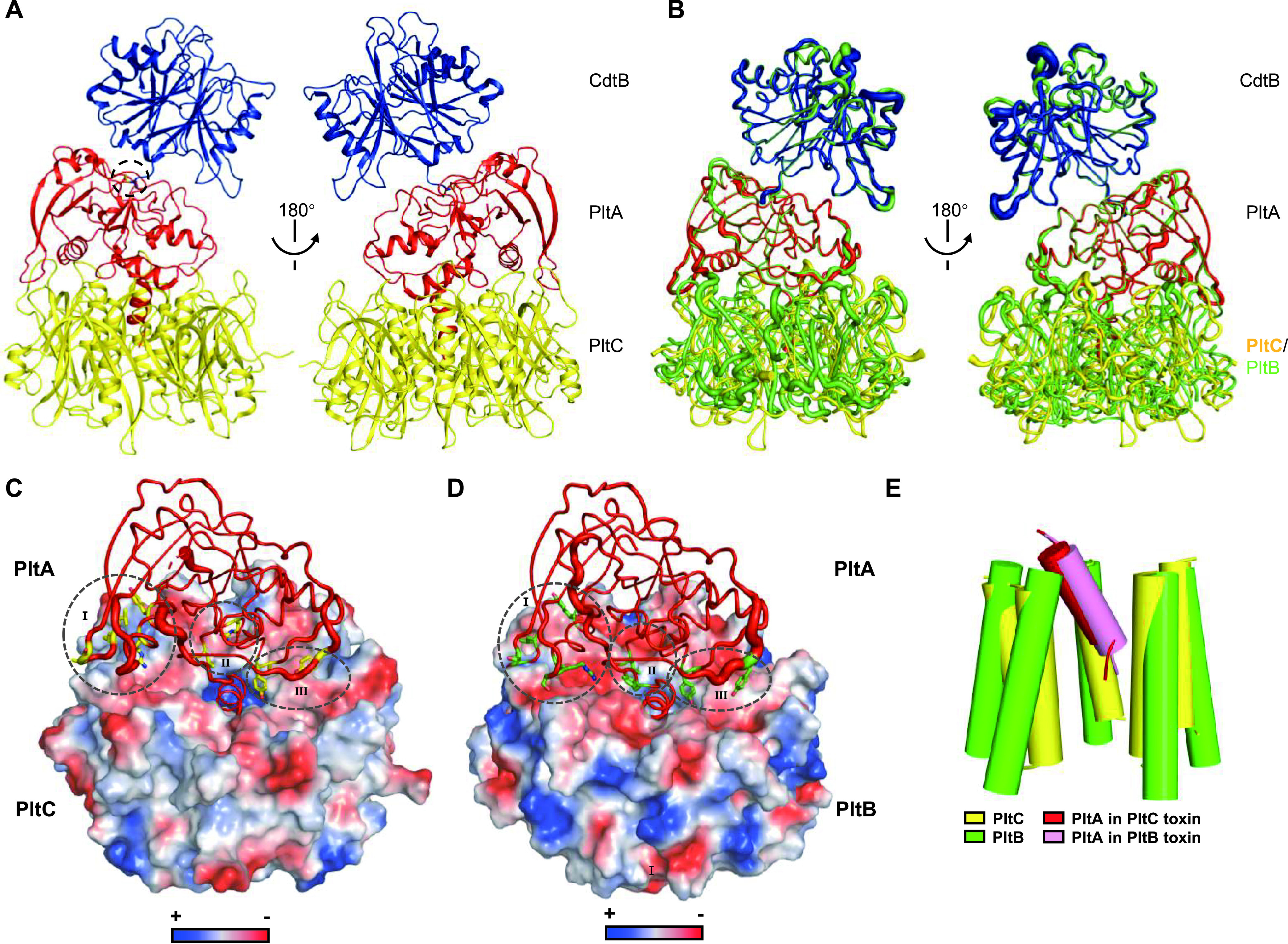
The structure of PltC typhoid toxin. (A) Two views of the overall structure of the PltC version of typhoid toxin shown as a ribbon cartoon and representing a 180° rotation about a vertical axis. The critical disulfide bond between CdtB and PltA is shown in stick form and highlighted with a dotted circle. CdtB, PltA, and PltC are shown in blue, red, and yellow, respectively. (B) Structure superimposition of PltC typhoid toxin (colored as in panel A) and PltB typhoid toxin (green) shown in two views. (C and D) Interfaces between PltA and PltC (C) and between PltA and PltB (D). PltA is shown as cartoon putty and the B subunits are shown using an electrostatic potential surface view. The amino acid residues from PltA that form interactions with the apical surfaces of the B subunits are shown using sticks and grouped into three different regions (I to III) highlighted with dotted circles. (E) The C-terminal helix of PltA inserts into the channel at the center of the B pentamer in a similar fashion in the PltB and PltC typhoid toxins. The α1 helix that defines the central pore of the PltC pentamer is shown as a yellow cylindrical cartoon, and the PltA C-terminal α-helix is shown in red; similar structural features from the structure of the PltB typhoid toxin are shown using green (PltB) and pink (PltA).

To gain insight into the structural basis for the organization of PltC typhoid toxin, we determined the crystal structure of PltC-PltA-CdtB holotoxin at 2.29 Å resolution (Fig. 3A and Table S1). Similar to the PltB typhoid toxin, PltC typhoid toxin also displays a pyramid-shaped architecture with the PltC homopentamer at the bottom and the two A subunits (CdtB-PltA) sitting on top. As in the PltB typhoid toxin, CdtB does not directly interact with the delivery platform and is tethered to the toxin primarily through the disulfide bond it forms with PltA (9) (Fig. 3A). Interestingly, the PltA and CdtB subunits sit atop the delivery platform in a nearly identical fashion in both typhoid toxins; PltA and CdtB align with an RMSD of 0.292 Å over 383 Cα atoms in the structures of the two typhoid toxins (Fig. 3B). Despite the fact that PltC and PltB share very little sequence identity, their interactions with PltA are strikingly similar. Although the detailed interactions differ, conserved regions of PltA interact with the apical surfaces of both PltC and PltB, which adopt similar shapes and have similar charge distributions (Fig. 3C and D and Fig. S4). The most salient feature of the A-B interactions of AB_5_-type toxins is that of the C-terminal helix of the A subunit, which is inserted into a pore at the center of the B subunit pentamer. In line with the global structural similarities of the PltC and PltB toxin, the C-terminal helix of PltA is inserted into the central pore in a manner that is overall similar in both typhoid toxins (Fig. 3E).

10.1128/mBio.01916-21.2 FIG S2 Interacting amino acids at the interface of the PltB homodimer. The insets of the blue box (A) and light blue box (B) represent the interacting amino acids in the upper part and lower part of the PltB dimer, respectively. Interacting residues are shown as sticks; interactions are shown as black dashes. Download FIG S2, PDF file, 0.2 MB. Copyright © 2022 Liu et al. 2022 Liu et al. https://creativecommons.org/licenses/by/4.0/ This content is distributed under the terms of the Creative Commons Attribution 4.0 International license.

10.1128/mBio.01916-21.3 FIG S3 Interacting amino acids at the interface of the ArtB homodimer. The insets of the blue box (A) and light blue box (B) represent the interacting amino acids in the upper part and lower part of the ArtB dimer, respectively. Interacting residues are shown as sticks; interactions are shown as black dashes. Download FIG S3, PDF file, 0.2 MB. Copyright © 2022 Liu et al. 2022 Liu et al. https://creativecommons.org/licenses/by/4.0/ This content is distributed under the terms of the Creative Commons Attribution 4.0 International license.

10.1128/mBio.01916-21.4 FIG S4 The interfaces between PltA and different B subunits. (A) The interacting amino acids at the interface of PltA and different B subunits. The insets represent the interacting amino acids involved in the interface of PltA-PltC and PltA-PltB, respectively. The interacting residues are shown as sticks. The residues involved in the hydrophobic interactions are in red (PltA), yellow (PltC), and green (PltB). The residues involved in the polar interactions are in magenta (PltA) and cyan (PltC and PltB). The residues from PltA are labeled in red. (B) The electrostatic potential surface view of the apical faces of PltC and PltB. The shape and charge distribution of apical surfaces of PltC and PltB are similar. Download FIG S4, PDF file, 0.2 MB. Copyright © 2022 Liu et al. 2022 Liu et al. https://creativecommons.org/licenses/by/4.0/ This content is distributed under the terms of the Creative Commons Attribution 4.0 International license.

Together, these results show that, despite the fact that PltC and PltB are only ∼31% identical at the sequence level, they produce typhoid toxins with highly similar overall structures.

### PltC outcompetes PltB for assembling typhoid holotoxin.

Although PltC and PltB use very similar structural scaffolds to assemble with PltA, the specific chemical interactions that hold together the A and B subunits are quite different in the two typhoid toxins. Notably, the pore radius of the channel at the center of the PltC pentamer is generally narrower than that of the PltB pore. Close inspection of the side chain interactions between the PltA C-terminal tail and two different B subunit channels shows that all five monomers from the PltC pentamer interact with the PltA C-terminal tail via 9 direct or water-mediated hydrogen bonds. In the PltB structure, however, only four monomers from the PltB pentamer interact with the PltA C-terminal tail via 7 direct or water-mediated hydrogen bonds (Fig. S5).

10.1128/mBio.01916-21.5 FIG S5 Interacting amino acids between PltA C-terminal tail and the two different B subunit channels. Interactions between PltA C-terminal tail and the PltC channel (top) and the PltB channel (bottom). Interacting residues are shown as sticks; interactions are show as black dashes. Download FIG S5, PDF file, 0.2 MB. Copyright © 2022 Liu et al. 2022 Liu et al. https://creativecommons.org/licenses/by/4.0/ This content is distributed under the terms of the Creative Commons Attribution 4.0 International license.

**FIG 4:**
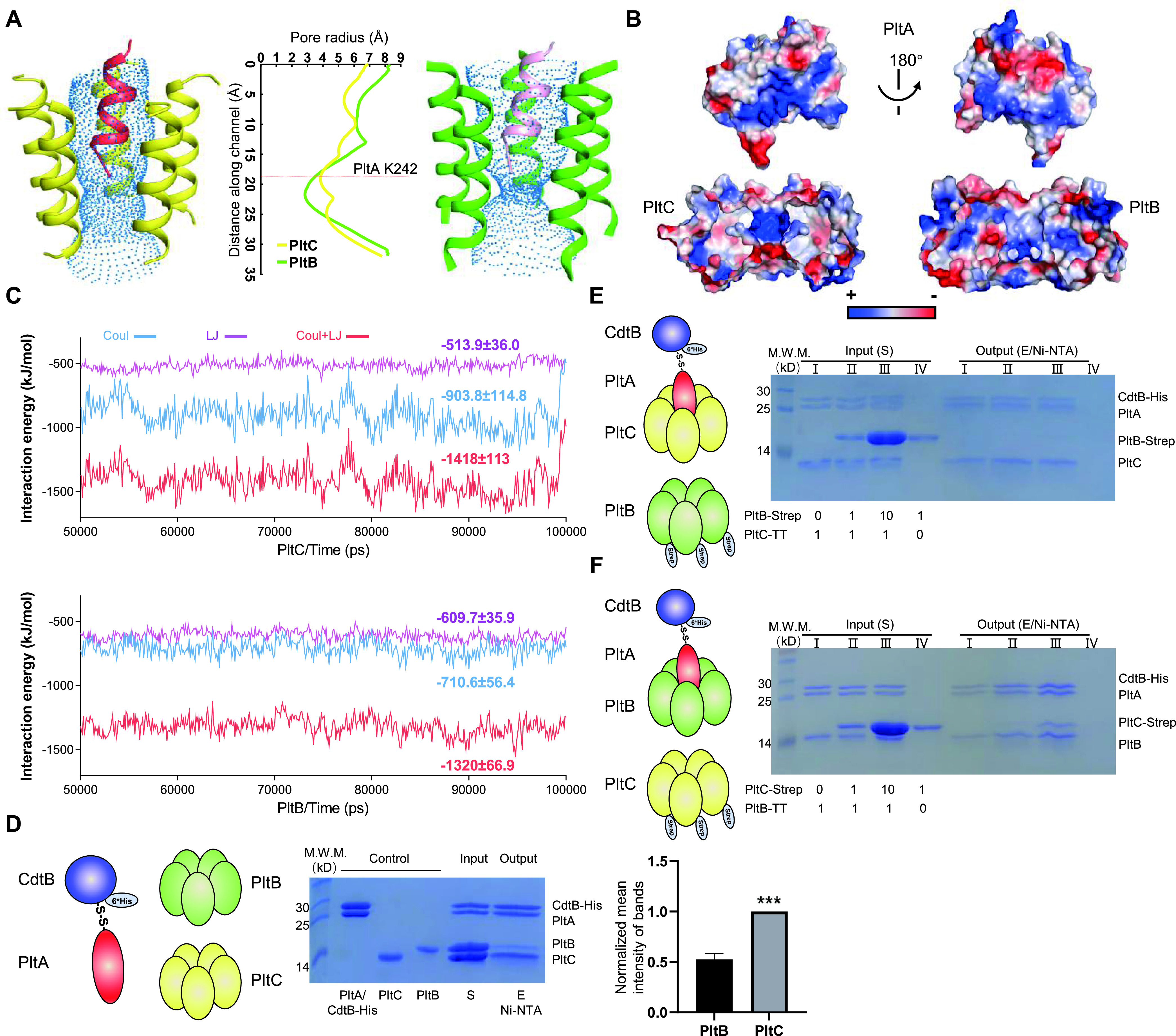
PltC outcompetes PltB for assembling into the typhoid holotoxin. (A) Comparison of the radius of the channels at the center of the PltC (yellow; left) and PltB (green; right) pentamers. The central graph shows the pore radius, calculated using the Hole program (coot software), along the length of the channel beginning at the apical face (where PltA inserts). The red dotted line in this graph shows where the tip of the PltA C terminus ends in the channels. (B) Two views of surface charge distribution of PltA, representing a 180° rotation about a vertical axis (top) and the electrostatic potential of the inner channels formed through PltC and PltB pentamers (bottom). (C) Interaction energy formed by PltA-PltC and PltA-PltB complexes, calculated using the last 50-ns trajectory of all-atom molecular dynamics simulations. Coul, electrostatic interactions; LJ, hydrophobic interactions. (D) B subunit competition assays. Purified PltA-CdtB-His_6_ was incubated with PltC and PltB at a molar ratio of 1:3:3 at room temperature for 5 h. The mixture (S) was then loaded on a Ni-NTA column, washed, and eluted (E). Samples were analyzed using 15% SDS-PAGE. The histogram shows the normalized mean intensity (with standard deviations [SD]) of the PltB and PltC bands from the eluted samples (E) in three independent experiments. ***, *P < *0.0005. (E and F) Competitive B subunit displacement assays. (E) The PltC holotoxin (PltC-PltA-CdtB-His_6_) and free pentameric PltB-Strep were mixed at molar ratios of 1:0, 1:1, 1:10, and 0:1 and incubated for 3 h at room temperature. The mixtures were then purified using Ni-NTA columns, and elution samples were analyzed using SDS-PAGE to examine whether PltB could replace PltC within the holotoxin. (F) An assay similar to that described for panel E was performed using PltB holotoxin (PltB-PltA-CdtB-His_6_) and purified PltC pentamer (PltC-Strep).

The charge distribution of the PltC and PltB channels is also very different in the upper part of the pore, the main region that interacts with the C-terminal α-helix of PltA; this region is highly positively charged in PltC and is more hydrophobic in PltB (Fig. 4B). An electrostatic surface potential analysis of the PltA C-terminal α-helix showed that one side is highly negatively charged, while the other side is relatively hydrophobic (Fig. 4B). Interestingly, this presents a likely scenario in which the major interactions between PltC and PltA are electrostatic, while the interactions between PltB and PltA are more hydrophobic. We employed all-atom molecular dynamics simulations to examine the interactions of PltA with PltC or PltB and found that the average electrostatic and hydrophobic interaction energy formed between PltA and PltC and between PltA and PltB were −1,418 ± 113 kJ/mol and −1,320.3 ± 66.9 kJ/mol, respectively (Fig. 4C). These calculations imply that PltA exhibits a stronger interaction with PltC than with PltB, which is largely due to the stronger electrostatic interactions between PltA and PltC (−903.8 ± 114.8 kJ/mol) compared to that between PltA and PltB (−710.6 ± 56.4 kJ/mol) (Fig. 4C). This energy difference in electrostatic interactions corresponds to ∼6 intramolecular hydrogen bonds (31) and is also consistent with our observation above that there are more hydrogen bonds between the PltA C-terminal tail and the PltC channel than between the PltA C-terminal tail and the PltB channel (Fig. S5). This is noteworthy given that previous experiments have indicated that there is an increase in the amount of PltB toxin that is produced by *S*. Typhi during infection in a Δ*pltC* strain compared to the wild type, suggesting that competition with PltC reduces the amount of PltB toxin formed during infection (23).

To explore the relative abilities of PltC and PltB to compete for A subunits to form typhoid toxin, we used an *in vitro* competitive binding assay to compare the ability of these B subunits to bind PltA-CdtB. We incubated equal amounts of purified untagged PltC and PltB with purified PltA-CdtB-His_6_ and then applied these mixtures to Ni-NTA columns and performed metal affinity chromatography (Fig. 4D). SDS-PAGE analysis showed that approximately 2-fold as much PltC was copurified with PltA-CdtB as PltB, indicating that PltC outcompeted PltB for PltA-CdtB (Fig. 4D). We further assessed B subunit competition using a second *in vitro* competition assay that assessed the ability of one B subunit to displace the other from the intact toxin. First, we mixed purified PltC toxin (featuring CdtB-His_6_) with purified PltB-Strep pentamer in different concentrations and performed metal affinity chromatography. SDS-PAGE analysis indicated that, even when present at a molar ratio of 10:1, PltB was unable to displace PltC from PltC toxin (Fig. 4E). However, a similar experiment wherein purified PltB toxin (CdtB-His_6_) was mixed with purified PltC-Strep pentamer showed significant displacement of PltB from PltB toxin by PltC at a 10:1 molar ratio (Fig. 4F). Together, these results show that PltC’s narrower pore and the stronger electrostatic interactions between this pore and the C-terminal helix of PltA enable PltC to outcompete PltB for assembling typhoid toxin.

### PltC and PltB exhibit different glycan binding specificities.

**FIG 5:**
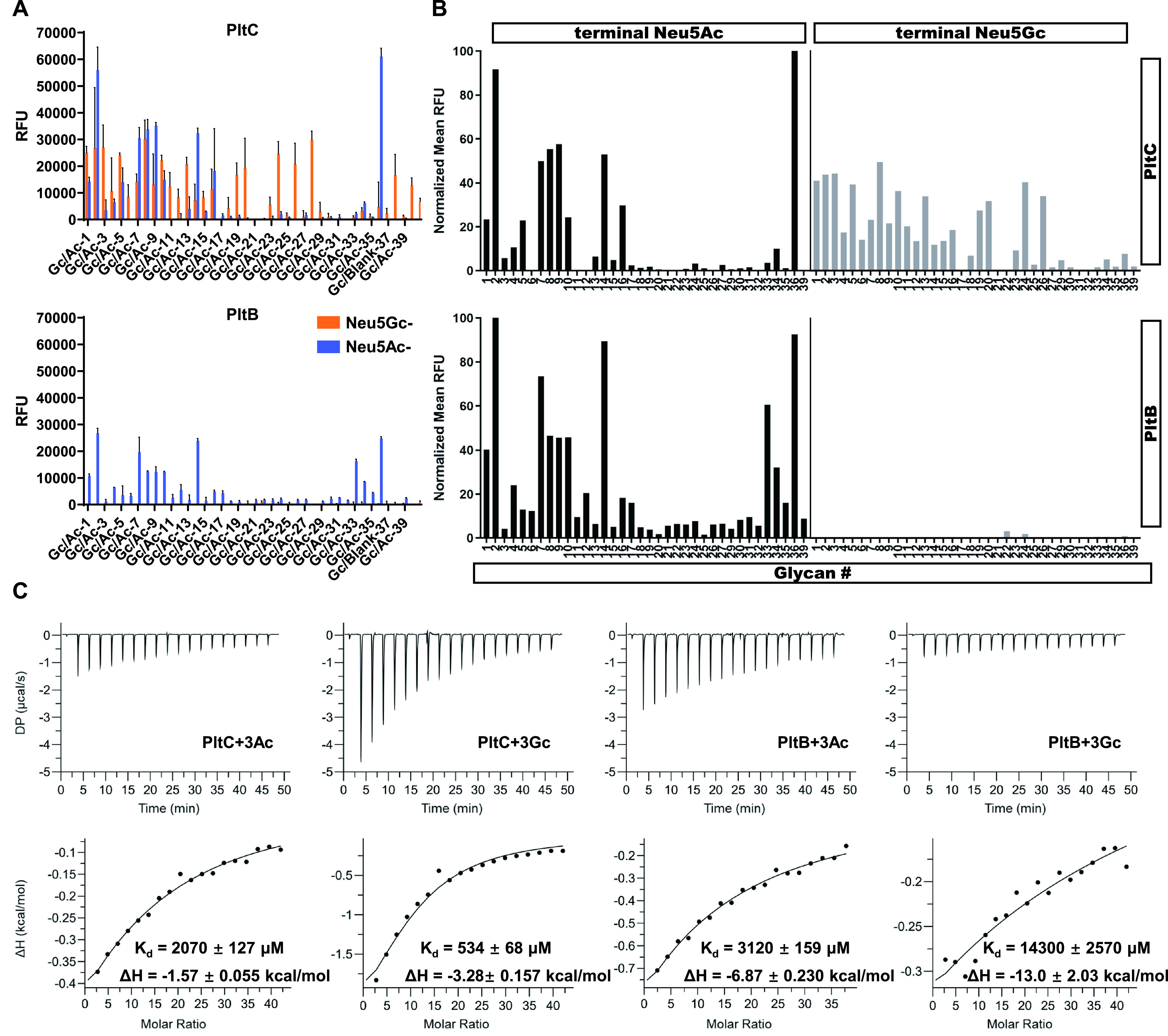
PltC and PltB exhibit different glycan binding preferences. (A) Glycan array analyzing glycan-binding preferences of PltC (top) and PltB (bottom). The *y* axis values represent averages and SD of the relative fluorescence units (RFU) from three independent measurements, and the *x* axis shows the glycan numbers from the array (Table S2A). (B) Comparison of PltC binding (top) and PltB binding (bottom) to paired Neu5Ac- and Neu5Gc-terminated glycans. The *y* axis values represent the normalized average RFU from three independent measurements, and the *x* axis indicates glycan numbers (Table S2C). (C) The binding affinities of Neu5Acα2-3Galβ1-3Glc (3Ac) and Neu5Gcα2-3Galβ1-3Glc (3Gc) to PltB and PltC were measured by ITC. The N values were fixed to 5 for PltB and 10 for PltC during the data fitting. Due to the low binding affinity between the typhoid toxin delivery subunits and glycans, the *K_d_* and ΔH values shown here may be inaccurate. These experiments were carried out at least three times with consistent results.

The PltB and PltC versions of typhoid toxin have previously been shown to exhibit substantial functional differences, but the mechanisms underlying these differences are not understood (23). Since the functional characteristics of the B subunits of AB_5_-type toxins are largely driven by their glycan binding properties, we used glycan array screening to compare the receptor binding specificities of PltB and PltC. This customized glycan array was composed of 37 pairs of sialylated glycan chains, where each pair represented an otherwise-identical glycan terminating in the two different sialic acid (Sia) forms, Neu5Ac and Neu5Gc. The array, designed to mimic glycans present on human cell surface receptors, followed the consensus structure Siaα2-3/-6Galβ1-4GlcNAc, which was previously reported as the favored binding sequences of PltB and ArtB (9, 10, 20). In general, we observed that both B subunits showed a stronger binding preference for biantennal N-glycans with a Neu5Acα2-6 linkage, compared to linear N-glycans (Fig. 5A and Table S2A). However, for N-glycans featuring a Neu5Acα2-3 linkage, both B subunits exhibited a higher affinity for certain linear sugars (Fig. 5A; Fig. S6; Table S2B), illustrating the complexity of glycan binding for bacterial toxins. Consistent with previous findings, we observed that PltB exhibits a strict specificity for sialoglycans terminated in Neu5Ac. In contrast, we observed that PltC binds both Neu5Ac- and Neu5Gc-terminated glycans and did not demonstrate a clear preference between the two sialic acids (Fig. 5A and B; Table S2C). This is in accordance with PltC’s close relationship with ArtB and previous work that has shown that ArtB is able to recognize both Neu5Ac- and Neu5Gc-terminated sialoglycans (10). To further evaluate the differences in PltB and PltC glycan binding specificities, we carried out an isothermal titration calorimetry (ITC) assay to measure the binding affinity between the two B subunits and the Neu5Ac/Neu5Gc-terminated versions of a representative glycan (Neu5Acα2-3Galβ1-4Glc and Neu5Gcα2-3Galβ1-4Glc). We found that PltC and PltB bound the Neu5Ac version of the glycan with similar affinity. Strikingly, however, PltC showed much greater affinity for the Neu5Gc glycan than its Neu5Ac-terminated counterpart, whereas the binding of PltB to the Neu5Gc derivative was almost undetectable (Fig. 5C). Together, these results show that PltB and PltC have noteworthy differences in their glycan binding specificities that are particularly evident with respect to the terminal sialic acid residue.

10.1128/mBio.01916-21.6 FIG S6 Complexity of glycan binding specificity of typhoid toxin B subunits. The glycan array analysis involves three groups: PltB binding to Neu5Ac-linked glycans, PltC binding to Neu5Ac-linked glycans, and PltC binding to Neu5Gc-linked glycans. Each group can be divided into two parts (α2-6-linked or α2-3-linked), and each part includes monoantennary glycans and biantennary glycans. For PltB/PltC binding to Neu5Ac groups, PltB or PltC probably binds biantennary glycans over monoantennary glycans in α2-6-linked *N*-acetylneuraminic acid, while in the α2-3-linked part, the situation is the opposite. However, for PltC binding to Neu5Gc groups, PltC binds preferentially to biantennary glycans in the α2-3-linked part, while in the α2-6-linked part, there is no obvious difference between PltC binding of biantennary and monoantennary glycans. Download FIG S6, PDF file, 0.10 MB. Copyright © 2022 Liu et al. 2022 Liu et al. https://creativecommons.org/licenses/by/4.0/ This content is distributed under the terms of the Creative Commons Attribution 4.0 International license.

10.1128/mBio.01916-21.10 TABLE S2 (A) PltC and PltB-binding to a customized sialoglycan microarray. (B) Analysis of fine ligand specificity of PltC and PltB. (C) Analysis of ligand specificity of PltC and PltB. Download Table S2, DOCX file, 0.04 MB. Copyright © 2022 Liu et al. 2022 Liu et al. https://creativecommons.org/licenses/by/4.0/ This content is distributed under the terms of the Creative Commons Attribution 4.0 International license.

### Ligand-bound structures of PltC reveal the molecular bases for its binding specificity.

**FIG 6:**
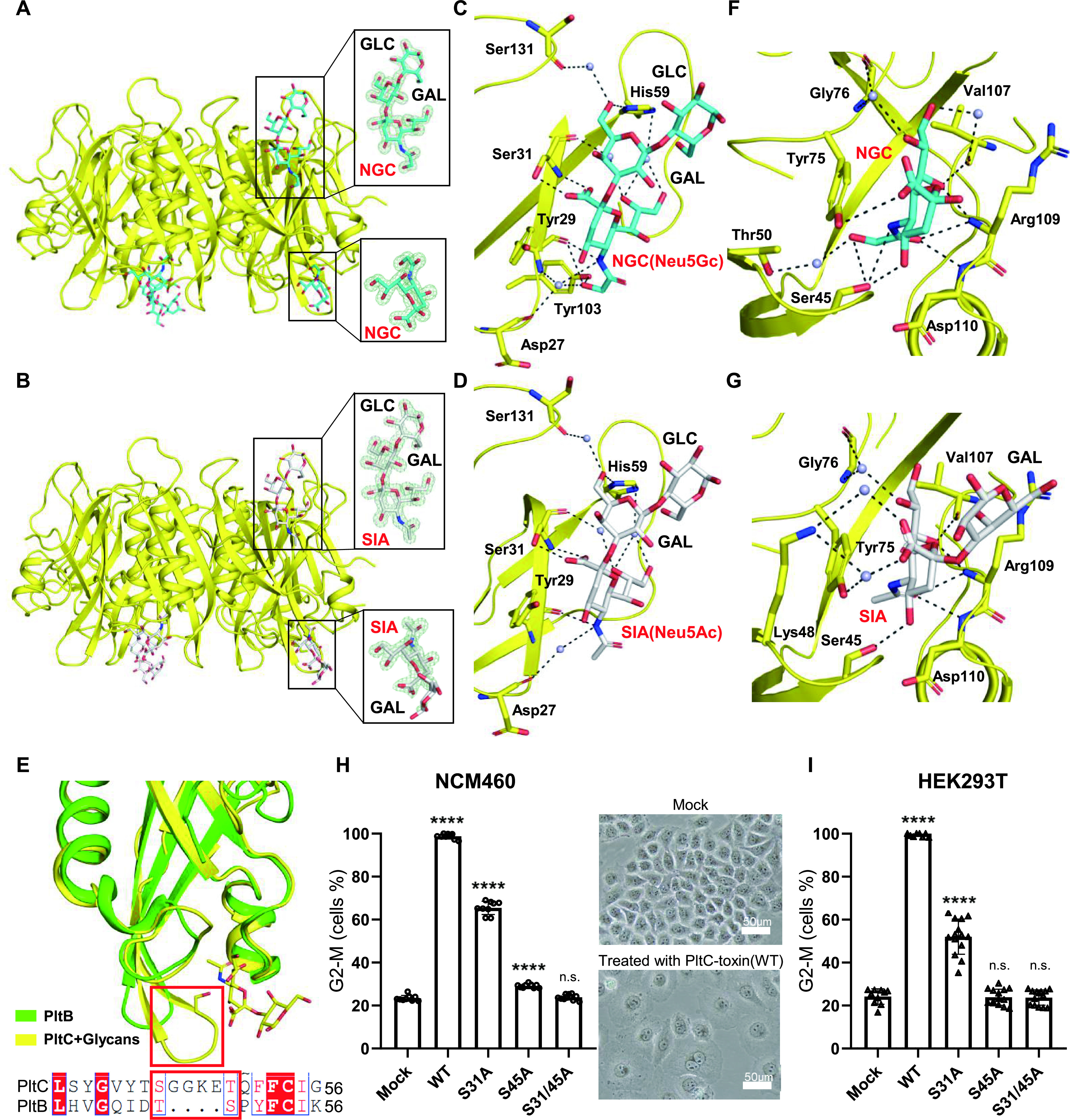
Glycan-bound atomic structures of PltC reveal that PltC has a second glycan binding pocket not found in PltB. (A) Crystal structure of PltC homopentamer in complex with the Neu5Gcα2-3Galβ1-4Glc shown as a ribbon cartoon. Cyan, blue, and red sticks represent carbon, nitrogen, and oxygen atoms in the sugar backbone. (Insets) Close-up views of Neu5Gcα2-3Galβ1-4Glc. (B) Crystal structure of PltC homopentamer in complex with the Neu5Acα2-3Galβ1-4Glc shown as a ribbon cartoon. White, blue, and red sticks represent carbon, nitrogen, and oxygen atoms in the sugar backbone. (Insets) Close-up views of Neu5Acα2-3Galβ1-4Glc. (A and B) Green mesh represents the sugar composite annealed omit difference density map contoured at 1.5σ. (C and D) The interactions between PltC and Neu5Gcα2-3Galβ1-4Glc (C) or Neu5Acα2-3Galβ1-4Glc (D) at the lateral binding site. (E) Structural comparison of PltC basal glycan binding site with the corresponding region of PltB. Blue and red sticks in the sugar backbone represent nitrogen and oxygen atoms, respectively. The amino acid sequence alignment of the relevant regions of PltC and PltB is shown at the bottom. The red box highlights the inserted sequence that is uniquely present in PltC that forms an integral part of this binding pocket. (F and G) The interactions between PltC and Neu5Gcα2-3Galβ1-4Glc (F) or Neu5Acα2-3Galβ1-4Glc (G) at the basal binding site. In panels C to G, PltC is shown as a yellow ribbon cartoon, the sugar and amino acids interacting with the sugar are shown as sticks, the interactions are shown as black dashes, and water is shown as light blue balls. (H and I) The toxicity of the PltC^S31A^, PltC^S45A^, PltC^S31/S45A^, and wild-type versions of the PltC toxin was assessed using cultured NCM460 cells (H) and HEK293T cells (I). The toxin concentrations used were 200 pM (H) and 25 pM (I). The percentage of cells arrested at the G_2_/M phase of the cell cycle was assayed using flow-cytometric analysis. Data points are means and SD from at least three independent experiments. ****, *P < *0.0001; n.s., not significant (*P > *0.05) compared to untreated cells. Representative light microscope images of untreated and PltC-typhoid-toxin treated NCM460 cells are shown in panel H. Bars, 50 μm.

To gain insight into the structural bases for PltC glycan-binding specificity, we solved the structure of PltC in complex with analogous glycans terminating with either Neu5Ac (Neu5Acα2-3Galβ1-4Glc, 1.40 Å resolution) or Neu5Gc (Neu5Gcα2-3Galβ1-4Glc, 1.24 Å resolution) (Fig. 6A and B; Table S1). As has been observed previously for PltB and ArtB, the structures of the apo and the glycan-bound forms of PltC are overall quite similar, indicating that glycan binding does not trigger large-scale conformational changes (Fig. S7). Both structures reveal that each PltC monomer displays the canonical lateral side glycan-binding site observed in other homologous B subunits, including PltB. Many of the amino acid residues that contact the glycan at this site in PltC are similar to those observed previously for PltB, such as amino acid residues Asp27, Tyr29, Ser31, and His59 (Fig. 6C and D). However, Tyr103 from PltC, which is absent in PltB, forms a direct hydrogen bond with the extra hydroxyl group of Neu5Gc (Fig. 6C) and provides a structural basis for PltC to bind both Neu5Ac- and Neu5Gc-terminated glycans. Interestingly, the PltC lateral binding site lacks key features observed in the PltB site that could weaken its glycan binding affinity. Specifically, PltB Tyr34, which forms π-π interactions with the glycan ring structure in PltB, is missing in PltC, and PltB Lys59, which forms a direct hydrogen bond with Neu5Ac and was reported to play a key role to glycan binding in PltB (20, 24), is replaced by histidine in PltC and forms much weaker water-mediated hydrogen bonds with glycans (Fig. 6C and D). Importantly, in addition to the lateral site, each PltC monomer has a second glycan-binding site located at the basal side of the protein (opposite from the face that interacts with PltA), which is formed by an additional spoon-like structure that is absent from PltB but which has also been observed in ArtB (10) (Fig. 6A, B, and E). In both the Neu5Acα2-3Galβ1-4Glc- and the Neu5Gcα2-3Galβ1-4Glc-bound PltC structures, the glycan is coordinated within this site through interactions with a number of residues, including Tyr75, Gly76, Val107, Arg109, Asp110, and others (Fig. 6F and G). The most critical residue within the second binding site is Ser45, which makes multiple contacts with both glycans and also stabilizes the extra hydroxyl group of the Neu5Gc-terminated glycan (Fig. 6F and G). The additional hydrogen bonds between PltC and the OH group that differentiates Neu5Gc from Neu5Ac are likely what confers the stronger binding affinity of PltC for Neu5Gcα2-3Galβ1-4Glc compared to its Neu5Ac derivative (Fig. 6C, D, F, and G).

10.1128/mBio.01916-21.7 FIG S7 Comparison of the atomic structures of PltC in different states. (A and B) Comparison of the atomic structures of the glycan-bound and apo forms of PltC. Binding to glycan receptors does not result in marked conformational changes in PltC. (C) Structural comparison between Neu5Ac-bound PltC and Neu5GC-bound PltC. Binding to different glycan receptors does not result in marked conformational changes in PltC. PltC is shown as a ribbon cartoon. The carbon atom of Neu5Gcα2-3Galβ1-3Glc is cyan and Neu5Acα2-3Galβ1-3Glc is white. The nitrogen and oxygen atoms in glycans are shown as blue and red sticks, respectively. Download FIG S7, PDF file, 0.2 MB. Copyright © 2022 Liu et al. 2022 Liu et al. https://creativecommons.org/licenses/by/4.0/ This content is distributed under the terms of the Creative Commons Attribution 4.0 International license.

To further investigate the glycan-binding capabilities of PltC, we analyzed the activities of purified PltC typhoid toxins—wild type and those carrying mutations to key residues within the glycan binding sites—in cellular intoxication assays. Specifically, we utilized the well-established activity assay which monitors the levels of G_2_-M cell cycle arrest in cultured cells, a cellular response to the CdtB-induced DNA damage elicited by typhoid toxin. In accordance with previous observations, when applied to the human epithelial cell lines NCM460 and HEK293T, the PltC toxin induced almost universal cell cycle arrest, as well as the overt cellular distension that is characteristic of CdtB intoxication (Fig. 6H and I). Compared to wild-type toxins, toxins carrying point mutations of key residues within either the lateral glycan binding site (S31A) or the basal site (S45A) both exhibited reduced activity against this cell line, indicating that both binding sites play functional roles for PltC (Fig. 6H and I). Notably, we found that the S45A mutation had a much more pronounced effect on the activity of PltC typhoid toxin than did the S31A mutation in both cell lines and, in fact, was almost as disruptive to toxin activity as the S31/45A double mutation (Fig. 6H and I). This is consistent with our observation that the lateral binding site in PltC is missing key interactions observed in the PltB site and suggests that the second (basal) binding site that is not found in PltB is the dominant glycan binding site for PltC. To further evaluate the roles of PltC’s two binding sites, we conducted ITC binding assays comparing the affinities of purified S31A, S45A, and S31A/S45A PltC for Neu5Gcα2-3Galβ1-4Glc and Neu5Acα2-3Galβ1-4Glc (Fig. S8). In accordance with the results above, we found that the S31A mutant exhibited binding affinities similar to that of wild-type PltC, whereas the binding affinities of the S45A mutant were drastically reduced and similar to those of the S31A/S45A double mutant. Together, these results indicate that PltC’s glycan binding specificity is largely mediated by a second glycan binding site not found in PltB, which is likely a key determinant of the different trafficking and cell targeting properties observed for the two typhoid toxins.

10.1128/mBio.01916-21.8 FIG S8 Glycan binding ability of different PltC variants. Representative ITC thermograms and fitting curves of PltC^S31A^, PltC^S45A^, and PltC^S31/45A^ titrated with Neu5Acα2-3Galβ1-3Glc (3Ac) or Neu5Gcα2-3Galβ1-3Glc (3Gc), respectively. The glycan binding affinity of PltC^S31A^ is similar to that of the wild type, while PltC^S45A^ and PltC^S31/45A^ show almost undetectable glycan binding ability. The N values were fixed to 5 for PltB and 10 for PltC during the data fitting. Due to the low binding affinity between the typhoid toxin delivery subunits and glycans, the *K_d_* and ΔH values shown here may be inaccurate. Each experiment was repeated at least twice with consistent results. Download FIG S8, PDF file, 0.3 MB. Copyright © 2022 Liu et al. 2022 Liu et al. https://creativecommons.org/licenses/by/4.0/ This content is distributed under the terms of the Creative Commons Attribution 4.0 International license.

## DISCUSSION

In this study, we present biochemical and structural evidence indicating that *S*. Typhi’s two typhoid toxin delivery subunits form distinct pools of structurally similar but functionally distinct toxins; this phenomenon is unique among AB_5_-type toxins characterized to date. This utilization of multiple distinct delivery subunits contrasts with that of pertussis toxin, which produces a single version of the toxin that contains four distinct B subunits in a heteropentameric delivery platform (14, 32). It is possible that *S*. Typhi’s production of two distinct toxins is an early step on the evolutionary path to producing a more complex, heteromeric delivery platform. However, another explanation for this phenomenon is that producing distinct pools of toxin with different trafficking and cell targeting properties is beneficial to the bacterium. It has been shown that the expression of *pltC* is controlled by a different regulatory network than the other typhoid toxin genes and that different environmental cues influence the expression of *pltC* and the other typhoid toxin genes (23). This suggests that *S*. Typhi is able to tune the amounts of the two different toxins in response to its environment, such that within different cell types or under different environmental conditions, *S*. Typhi may produce different toxins with different glycan binding specificities to execute specific tasks. It is also noteworthy that it has been shown that PltC typhoid toxin produced during infection of cultured human epithelial cells remains associated with the SCV during infection, while the PltB toxin is exocytosed (23). Producing a pool of toxin that remains associated with the SCV could potentially be beneficial to the bacterium. For example, toxin that has accumulated with the SCV would be released upon the death of the infected host cell, potentially inactivating surrounding immune cells and thus protecting *S*. Typhi from detection by the host immune system.

A recent study of the typhoid toxins produced by Salmonella enterica serovar Javiana found that the PltB and PltC homologs present in this serovar both preferentially form homomeric interactions (26). Under certain experimental conditions, however, this study also found evidence for weak interactions between PltB and PltC, the relevance of which is not yet clear (26). The data presented here indicate that *S*. Typhi typhoid toxins with PltB/PltC heteromeric delivery platforms do not assemble and also provide a structural basis for this finding. These data are consistent with the findings of a previous study that explored these interactions in their native context in *S*. Typhi both grown *in vitro* and isolated from infected human cells (23). Although the sequence identity between *S*. Typhi PltB and PltC and the homologs found in *S*. Javiana is high (both proteins are ∼95% identical between the two serovars), there are several amino acid differences located around the interfaces between the monomeric B subunits that may explain the observed differences. Further studies will be required to uncover the mechanisms and outcomes of typhoid toxin assembly from different S. enterica serovars.

The similar assembly of the two different typhoid toxins is a remarkable example of “Lego-like assembly,” where a conserved connection scaffold enables very different components to assemble in a similar fashion. PltB and PltC exhibit significantly different structural and functional characteristics, yet their basic mechanisms of interacting with PltA are quite similar, yielding structurally analogous toxins. Importantly, we show here that PltC is able to outcompete PltB to assemble typhoid toxin. This is consistent with previous results showing that there is an increase in the amount of PltB toxin that is produced during infection in a *pltC* deletion strain (23). PltC’s ability to outcompete PltB to form toxin is likely an important feature in order to produce the two pools of toxin, since PltB and PltA are cotranscribed (5) and are both secreted to the periplasm using the Sec system, and thus, PltB presumably benefits from a significant spatial-temporal colocalization with PltA that would otherwise present a significant barrier to PltC toxin formation. Although regulatory mechanisms that operate post-transcriptional initiation could conceivably unhinge PltA and PltB production, to the best of our current knowledge the production of PltA and PltB is coupled. The regulation of *pltC*, which is transcribed separately using distinct regulatory factors, would therefore represent the key factor in dictating the relative abundance of the two typhoid toxins. Further exploring the conditions and regulatory factors that control *pltC* expression could therefore offer important clues into the environments in which the different typhoid toxins are preferentially deployed.

The different properties and functions of the PltB and PltC toxins are presumably centered on their glycan-binding properties. We show here that the two typhoid toxins exhibit notable differences in their glycan binding properties, most significantly at the terminal sialic residue. The AB_5_ arrangement for bacterial toxins is proposed to offer the advantage that each toxin complex has multiple receptor binding sites that could potentially operate synergistically by simultaneously engaging neighboring glycans to increase the affinity of the toxin for its cognate receptor(s). In this context, it is noteworthy that the dominant glycan binding sites of PltB and PltC are located on different faces of the pentamer, and thus, the relative spatial arrangements of the different glycan binding sites on the two toxins are completely different. This difference could significantly impact the capacity of these toxins to simultaneously engage multiple glycans in a manner that would hinge on the spatial arrangement of the specific target receptors on the cell surface. In this manner, it is possible that the different positioning of the primary glycan-binding sites on the PltC and PltB toxins could potentially be a major factor in dictating the cells type(s) and tissue(s) targeted by these toxins during infection.

In this paper, we present atomic-level insights into how *S*. Typhi utilizes two different delivery subunits to assemble functionally diverse typhoid toxins. This study sheds light on important and poorly understood aspects of the remarkably complex biology of this toxin and has important implications for the development of preventative or therapeutic strategies that target typhoid toxin to combat typhoid fever.

## MATERIALS AND METHODS

### Plasmid construction.

The *pltB*, *pltC*, *pltA*, and *cdtB* genes were amplified from purified *S*. Typhi genomic DNA, and *artB* was amplified from *S.* Typhimurium DT104 genomic DNA. *pltB*, *pltC*, and *artB* were cloned into the pET28b (Novagen) vector with a C-terminal His tag or cloned into pET21d vector, which is derived from pET21b (Novagen) and includes the DrICE digestion site (which allows the removal of the C-terminal his tag) or cloned into the pACYC (Novagen) vector with a C-terminal Strep tag. Constructs featuring the full toxin (with a 6×His tag at the carboxy terminus of *cdtB*) were cloned by inserting each of the subunits as a single operon in pET28b or pET21d. Plasmids used in this study were constructed using Gibson assembly (33) and verified by DNA sequencing.

### Recombinant protein expression and purification.

Proteins for structural study were expressed in E. coli BL21(DE3) and grown in autoinduction Terrific broth (TB) at 25°C for 48 h. For the functional assays, smaller batches of proteins were prepared by expressing them in E. coli BL21(DE3) grown in LB medium to an optical density at 600 nm (OD_600_) of 0.8 at 37°C Expression was subsequently induced by addition of 0.4 mM IPTG (isopropyl-β-d-thiogalactopyranoside), and induced cultures were incubated overnight at 25°C. Bacterial cells were pelleted by centrifugation and then resuspended in lysis buffer (20 mM Tris-HCl, pH 8.0, and 150 mM NaCl) containing 100 μg/ml DNase, 100 μg/ml lysozyme, and 1 mM phenylmethylsulfonyl fluoride (PMSF). Bacteria were then lysed with a high-pressure cell disrupter (Union-Biotech, catalog no. UH-06), and clarified supernatants were obtained by centrifugation at 17,000 × *g* for 1 h. Target proteins with the His tag were purified using Ni-NTA columns (Qiagen, catalog no. 30230), using three washes with lysis buffer containing 20 mM imidazole and were eluted with lysis buffer containing 300 mM imidazole. Proteins with a DrICE enzyme digestion site in front of the 6×His tag were incubated with homemade DrICE protease for 3 h at room temperature in Ni-NTA columns and eluted with lysis buffer to remove the His tag. Proteins with a Strep tag were purified using columns filled with Strep-Tactin beads (Smart-lifesciences, catalog no. SAO53100), which were washed three times using lysis buffer and eluted with lysis buffer containing 2.5 mM desthiobiotin (Sigma, catalog no. D1411). Proteins were further purified by ion-exchange chromatography with a HiTrap S column (GE Healthcare, catalog no. 17115201) for holotoxins and a HiTrap Q column (GE Healthcare, catalog no. 17115401) for PltB, PltC and ArtB subunits. Fractions from the ion-exchange chromatography were analyzed using SDS-PAGE and further purified by Superdex 200 Increase (GE Healthcare, catalog no. 28990944).

### Crystallization.

The purification of 6×His-tagged PltC typhoid toxin used for crystallization is described above. Initial spare matrix crystallization trials of PltC holotoxin (5 mg/ml) were carried out with a crystallization robot (mosquito LCP; TTP Labtech). After several rounds of optimization, the PltC holotoxin (5 mg/ml) crystals grew in 3 days using the hanging drop vapor diffusion method in a mixture of 1 μl protein and 1 μl of reservoir solution consisting of 10% (wt/vol) polyethylene glycol (PEG) 10000, 0.1 M sodium citrate (pH 4.4), and 15% acetone. The C-terminal His tag of the purified PltC protein (7 mg/ml) was removed by DrICE enzyme digestion, and the untagged protein was used for initial crystal screening. Crystals were optimized using the hanging drop vapor method as described above and obtained under 34% (wt/vol) PEG 500 MME and 0.1 M sodium citrate, pH 5.0. Crystals appeared in 1 day, matured in ∼3 days, and were soaked with Neu5Acα2-3Galβ1-3Glc or Neu5Gcα2-3Galβ1-3Glc (separately) to get glycan-bound crystals. All crystals were soaked with 30% glycerol and flash-frozen in liquid nitrogen upon harvesting.

### X-ray data collection and structure determination.

X-ray diffraction data were collected on the BL17U1 and BL19U beamlines at the Shanghai Synchrotron Radiation Facility (Shanghai, China) and processed by HKL2000 (34). The apo and Neu5Ac- or Neu5Gc-bound PltC structures were determined by molecular replacement using the Phaser program in CCP4 (35) with the atomic coordinates of PltB (PDB code 4RHR), and the phase of PltC holotoxin was determined using the PltB typhoid toxin (PDB code 4K6L). Manual building was carried out in coot (36) to complete these models, and the structure refinement was done by Phenix (37). Figures were prepared using PyMOL (v.2.3.2 [https://pymol.org/]; Schrödinger, New York, NY, USA). The data collection and refinement statistics are summarized in Table S1.

### Molecular dynamics simulations.

The protein structures of the PltA-PltC complex from the PltC typhoid toxin (PDB code 7EE6) and of the PltA-PltB complex from the PltB typhoid toxin (PDB code 4K6L) were used in all-atom molecular dynamics simulations. For each simulation, the proteins were placed in a cubic box (10.6 by 10.6 by 10.6 nm) which was solvated with TIP3P water molecules and neutralized with 0.1 mol/liter NaCl. All simulations were carried out using Gromacs 2020 with a CHARMM36 force field. Energy minimization was performed using the steepest-descent algorithm. Six-step equilibration simulations were conducted by gradually turning off the restraints on the heavy atoms of proteins. Subsequently, 300-ns production simulations were conducted at 310 K and 10^5^ Pa. Periodic boundary conditions were considered. Temperature and pressure coupling was achieved via the Nose-Hoover and the Parrinello-Rahman methods, respectively. The LINCS method was used to constrain all bonds linking hydrogen atoms in proteins. The particle mesh Ewald (PME) method and Lennard-Jones potential algorithm were used to evaluate the electrostatic and hydrophobic interactions, respectively. The time step in production simulations was 2 fs.

### Cell lines and cell culture.

HEK293T cells were cultured in Dulbecco’s modified Eagle medium (DMEM; Gibco, catalog no. 8120010) containing 10% fetal bovine serum (FBS; BI, catalog no. 04-001-1ACS), NCM460 cells (human colonic epithelial cell line) were cultured in RPMI 1640 medium (Macgene, catalog no. CM10040) supplemented with 10% FBS. All cell lines were originally obtained from the ATCC and cultured at 37°C with 5% CO_2_ in a humidified incubator. These cell lines were routinely tested for mycoplasma contamination using a mycoplasma detection kit (Vazyme, catalog no. D101-01).

### Mammalian cell intoxication assay.

The toxicity of PltB and PltC toxins were assessed by analyzing the proportion of cells arrested in G_2_/M using flow cytometry as previously described (9). Briefly, cells were seeded in a 12-well plate at a density of ∼20% (5 × 10^4^ cells/ml for HEK293T and 2.5 × 10^4^ cells/ml NCM460). Twenty-four hours later, the medium was replaced with fresh medium containing toxins at the indicated concentrations. Cells were harvested when the cell density reached ∼70% (48 h for HEK293T and 72 h for NCM460). The harvested cells were pelleted and resuspended in 300 μl phosphate-buffered saline (PBS) and fixed by adding 700 μl prechilled ethanol and incubating at −20°C for >1 h. The fixed cells were pelleted, washed with PBS, and then incubated with PBS containing 50 μg/ml propidium iodide, 0.1 μg/ml RNase A, and 0.05% Triton X-100 for 30 min at 37°C. The stained cells were pelleted, washed, resuspended in PBS, filtered, and analyzed with a flow cytometer (BD, catalog no. C5). The DNA content of cells was determined using FlowJo software.

### Isothermal titration calorimetry.

To analyze the affinity between glycans and B subunits, isothermal titration calorimetry experiments were performed with a Malvern PEAKCal device. Stock solutions of 100 mM Neu5Acα2-3Galβ1-4Glc or Neu5Gcα2-3Galβ1-4Glc were diluted into 15 mM with ITC buffer containing 20 mM HEPES (pH 7.5) and 150 mM NaCl. C-terminally hexahistidine-tagged PltB and PltC were purified as described above and were then exchanged into ITC buffer with a Superdex 200 Increase column. For all titrations, 300 μl of 70 μM B subunit was titrated with 70 μl of glycans in 19 steps at 25°C. All samples were centrifuged before analysis with ITC. Evaluation of the titrations was carried out with the MicroCal evaluation software from Malvern (Worcestershire, UK).

### Glycan arrays.

Purified PltB and PltC were incubated with Cy3 reactive dyes for 1 h and dialyzed three times to remove free dyes from dye-protein conjugates. The glycan microarray slides were blocked for 30 min and incubated with fluorescently labeled proteins for 1 h in the dark. The glycan microarray slides were then washed to remove nonspecifically bound proteins and subjected to scanning with a microarray scanner (CapitalBio, catalog no. LuxScan-10K/A).

### Sequence alignment and phylogenetic analysis.

Amino acid sequence alignments of PltB and PltC from *S*. Typhi strain CT18 as well as ArtB from *S.* Typhimurium strain DT104 were done using ClustalX2, and the figures were generated using ESPript-3.0. Phylogenetic analysis of *S*. Typhi PltB and PltC and *S.* Typhimurium ArtB was inferred by using the maximum-likelihood method and conducted in MEGA X.

### Statistical analysis.

All statistical analyses were conducted using GraphPad Prism version 8.0 (GraphPad). All functional assays were performed at least three times independently. Two-tailed Student’s *t* tests were performed to determine the statistical significance for two-group comparisons. One-way analysis of variance (ANOVA) was used to determine the statistical significance for multiple comparisons. A *P* value of <0.05 was considered statistically significant.

### Data availability.

Coordinates for the atomic structures have been deposited in the RCSB Protein Data Bank under RCSB IDs 7EE3, 7EE4, 7EE5, and 7EE6. The data that support the findings of this study are available from the corresponding author upon reasonable request.
