# Supplementary figures and images for: Molecular Insights into the Assembly and Functional Diversification of Typhoid Toxin
Source: mBio. 2022 Jan 11;13(1):e01916-21. doi: 10.1128/mbio.01916-21 (PMC8749428; doi:10.1128/mbio.01916-21)

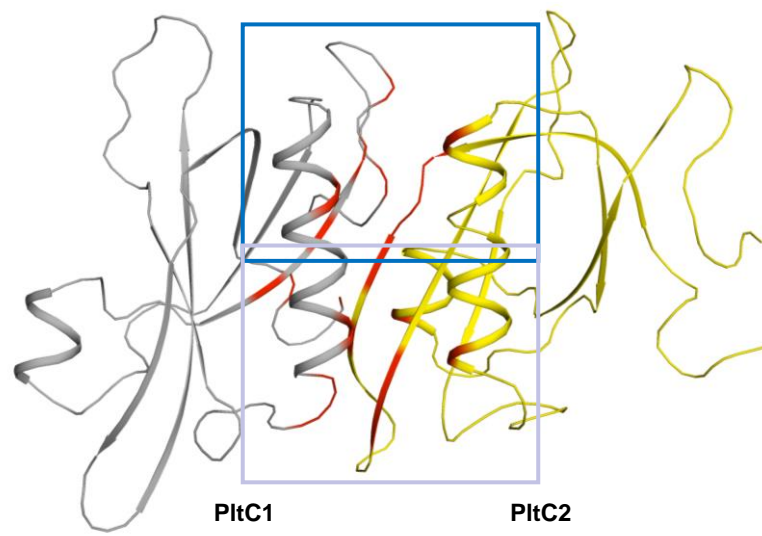

A

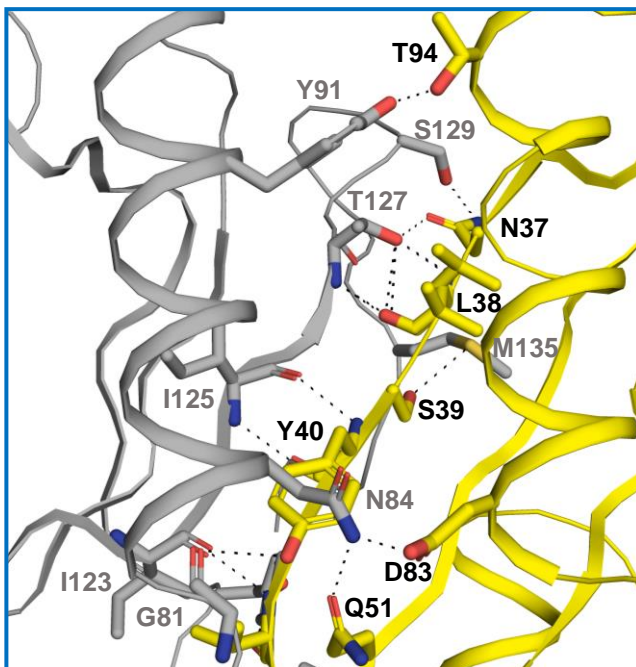

B

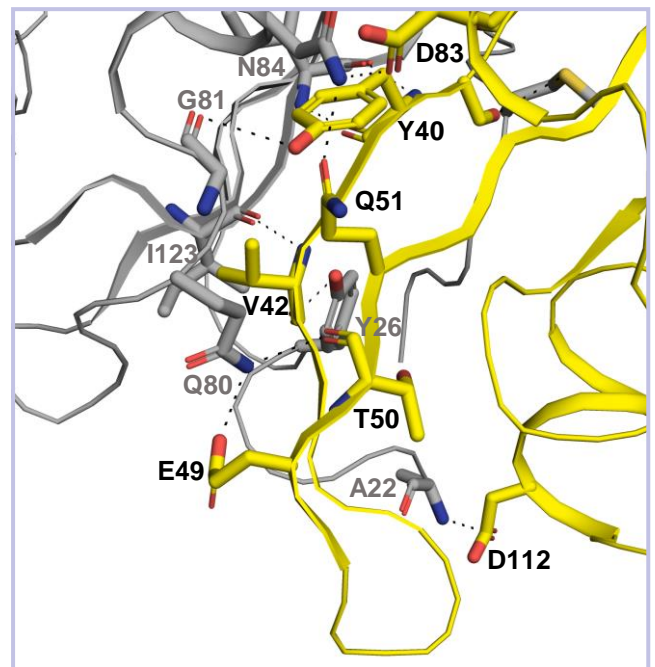

FIG S1

Supplement: FIG S1 [file mbio.01916-21-sf001.pdf]

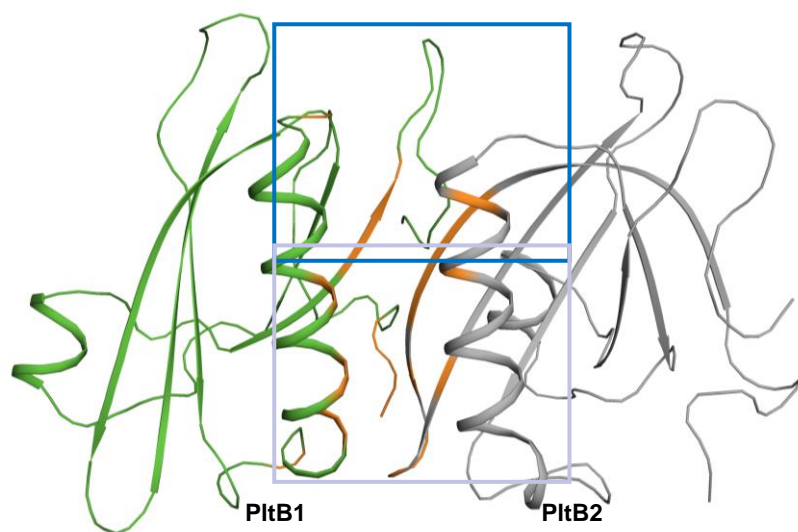

**A**

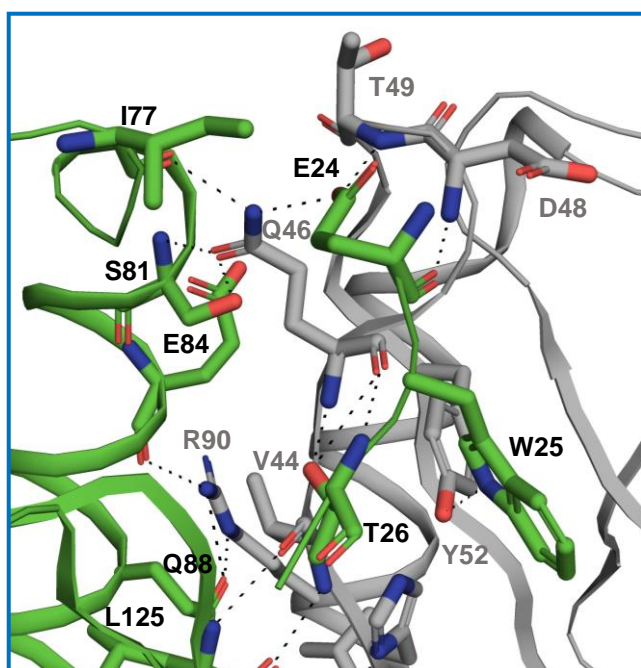

**B**

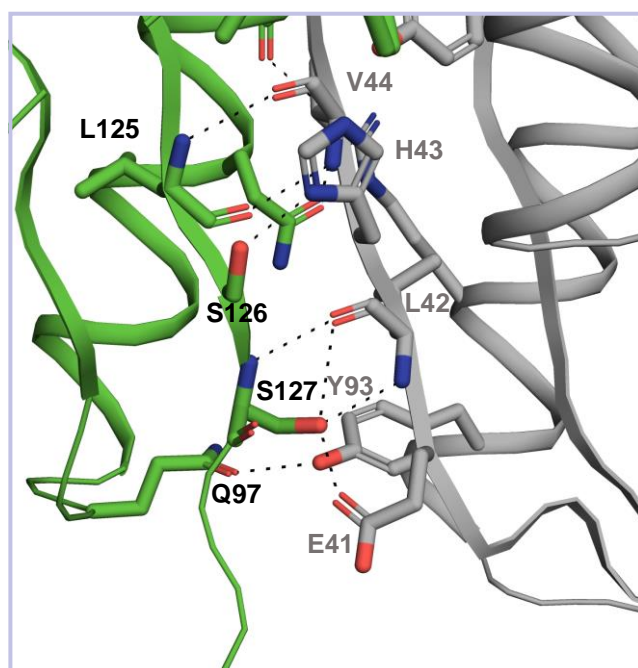

FIG S2

Supplement: FIG S2 [file mbio.01916-21-sf002.pdf]

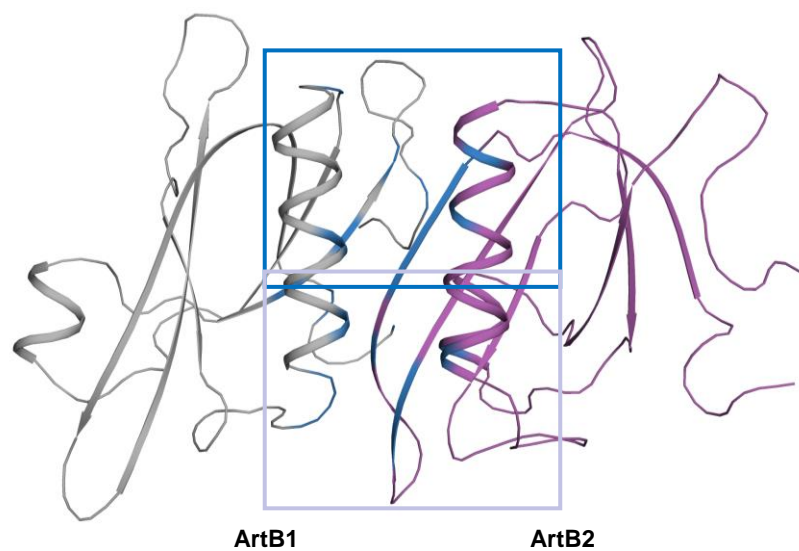

A

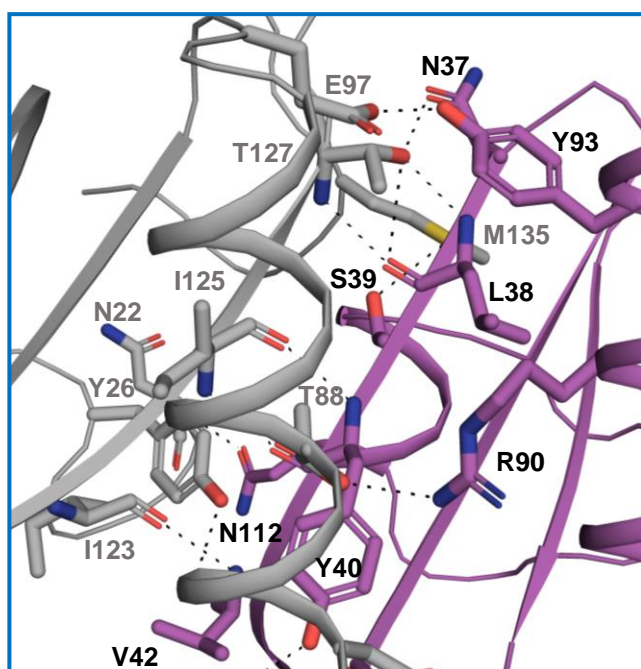

B

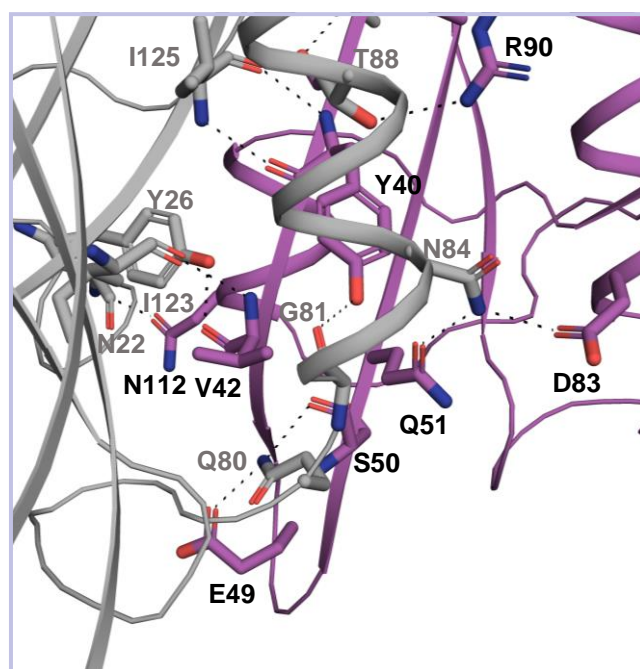

FIG S3

Supplement: FIG S3 [file mbio.01916-21-sf003.pdf]

**A**

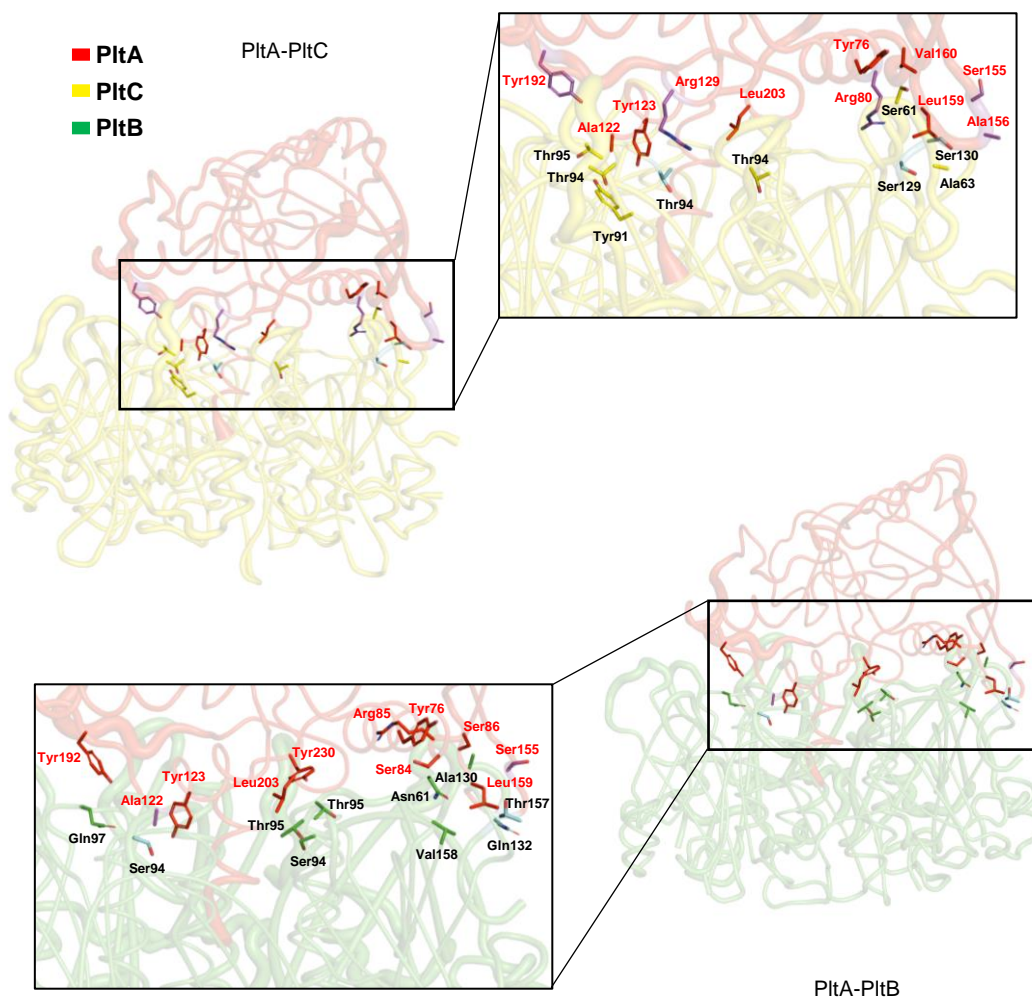

**B**

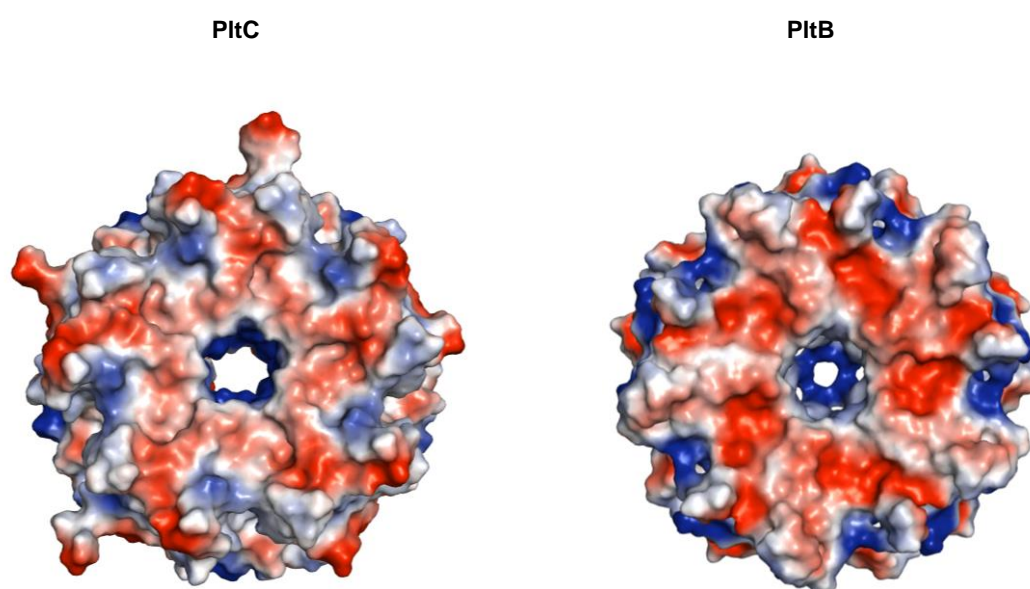

**FIG S4**

Supplement: FIG S4 [file mbio.01916-21-sf004.pdf]

# PltC toxin

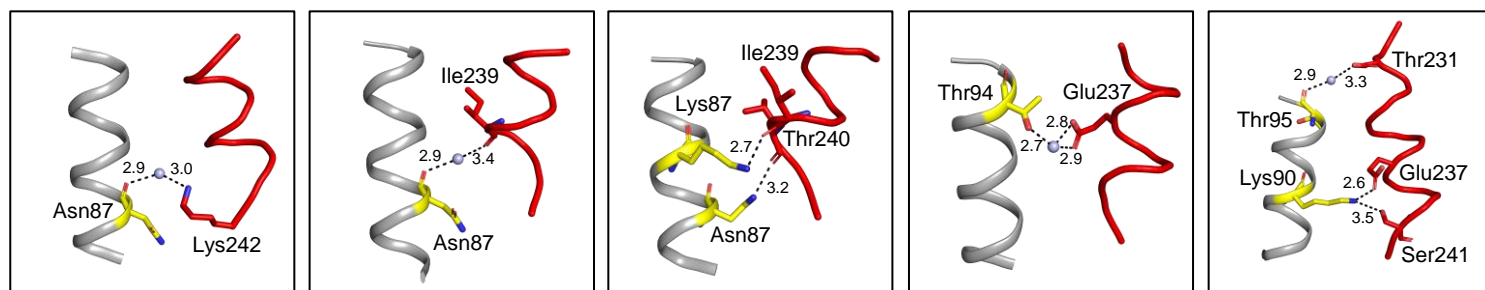

# PltB toxin

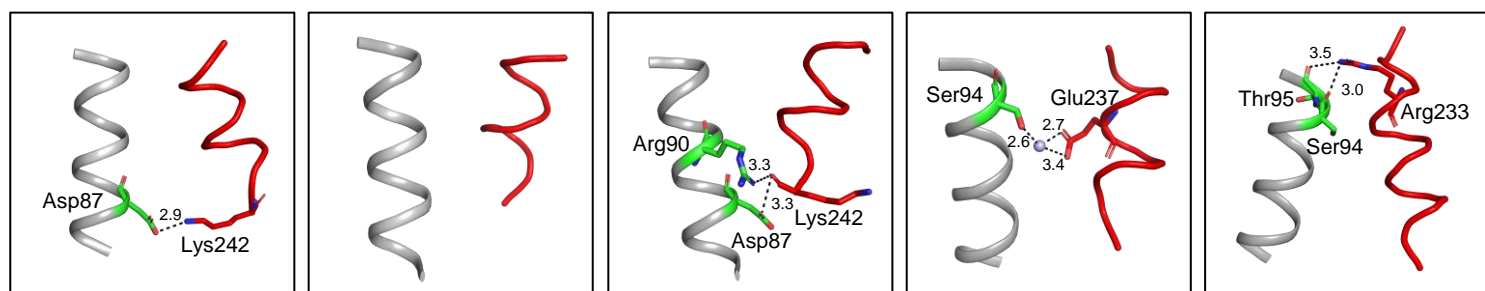

Chain A

Chain B

Chain C

Chain D

Chain E

FIG S5

Supplement: FIG S5 [file mbio.01916-21-sf005.pdf]

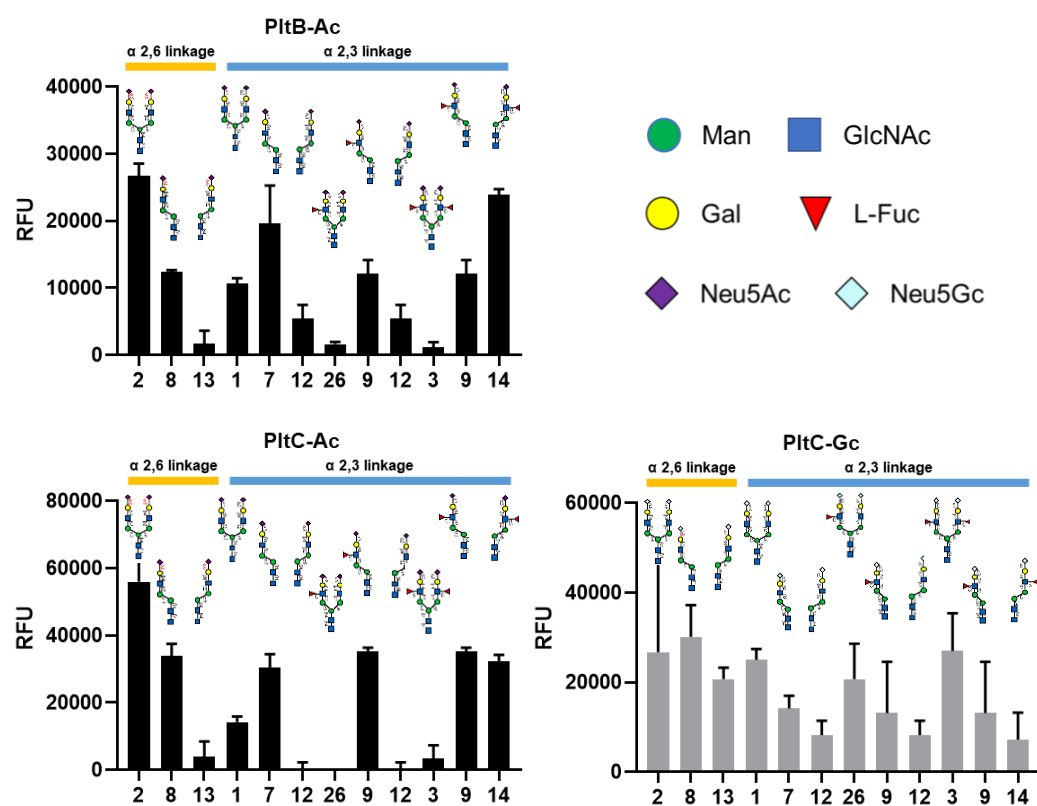

FIG S6

Supplement: FIG S6 [file mbio.01916-21-sf006.pdf]

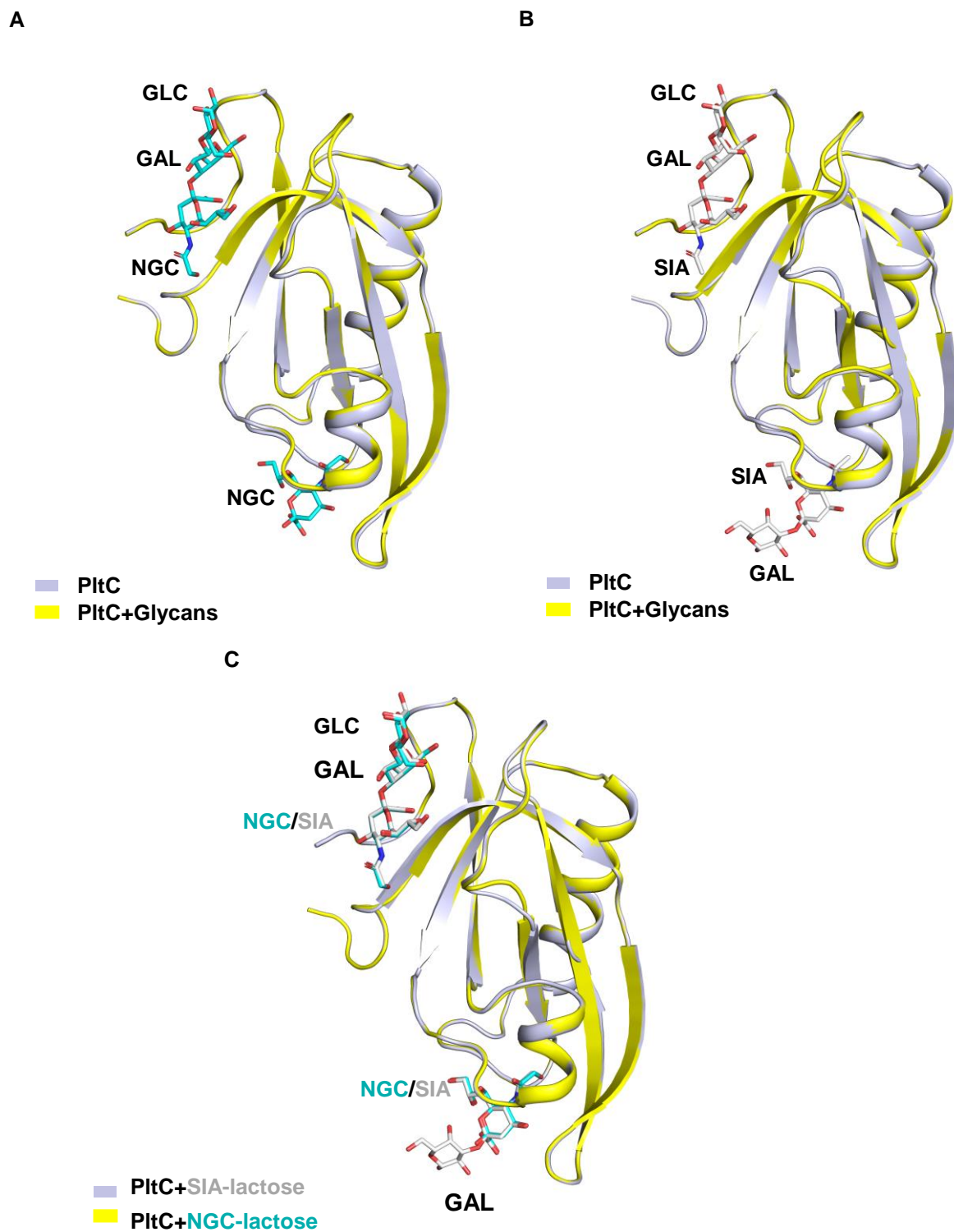

FIG S7

Supplement: FIG S7 [file mbio.01916-21-sf007.pdf]

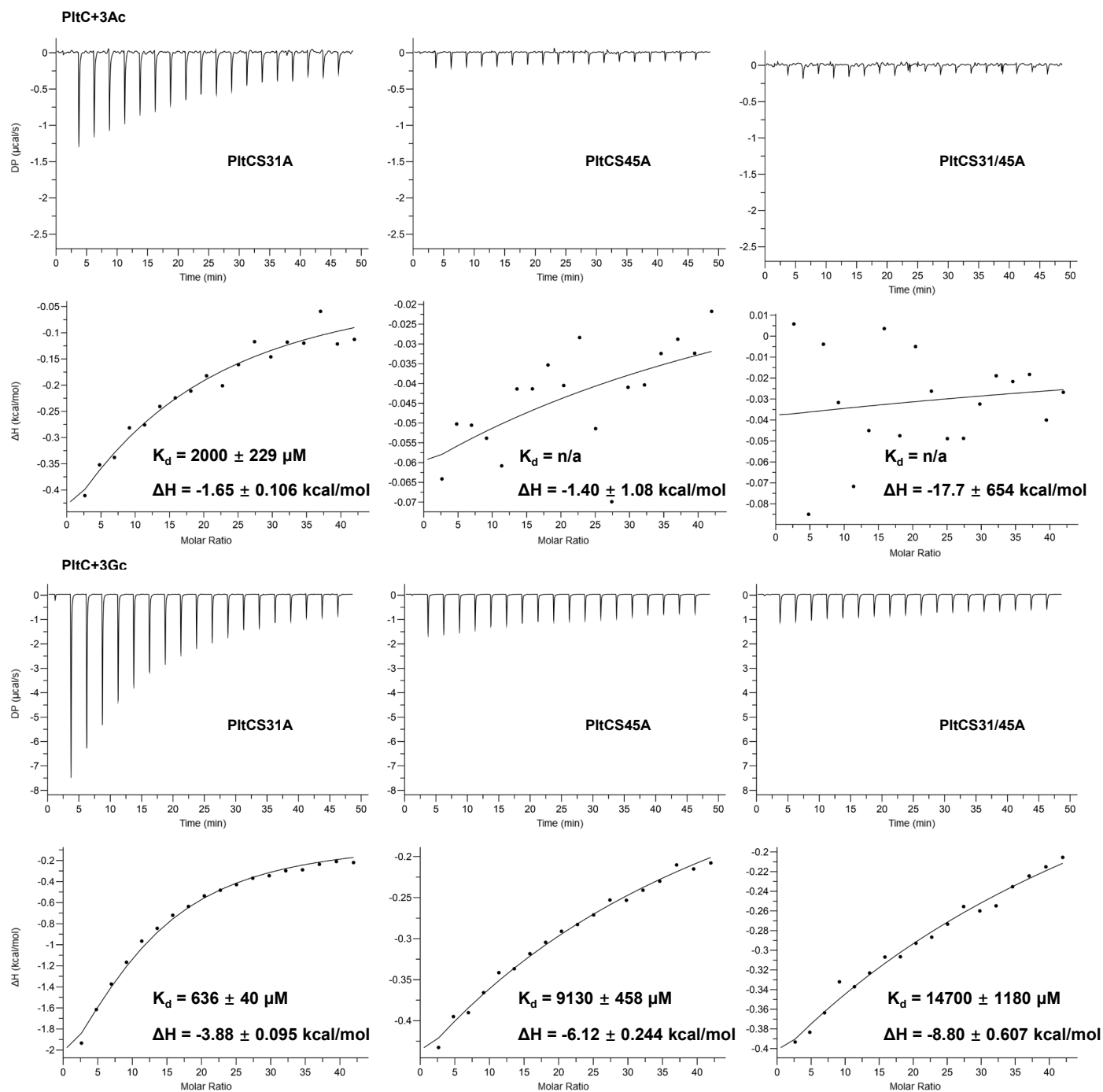

FIG S8

Supplement: FIG S8 [file mbio.01916-21-sf008.pdf]
