# Supplementary material for: Molecular Insights into the Assembly and Functional Diversification of Typhoid Toxin
Source: mBio. 2022 Jan 11;13(1):e01916-21. doi: 10.1128/mbio.01916-21 (PMC8749428; doi:10.1128/mbio.01916-21)
Supplement: TABLE S1 [file mbio.01916-21-st001.docx]

**TABLE S1. Data Collection and Refinement Statistics**

|  | PltC | PltC+Neu5Ac | PltC+Neu5Gc | PltCtoxin |
| --- | --- | --- | --- | --- |
| PDB ID | 7EE3 | 7EE4 | 7EE5 | 7EE6 |
| **Data collection** |  |  |  |  |
| Wavelength | 0.9793 | 0.9792 | 0.9785 | 0.9785 |
| Resolution range | 24.79 - 1.33  (1.378 - 1.33) | 27.89 - 1.40 、(1.45 - 1.40) | 27.29 - 1.24  (1.285 - 1.24) | 49.08 - 2.29  (2.372 - 2.29) |
| Space group | R 3 | R 3 | R 3 | P 21 21 2 |
| a b c | 189.607 189.607 41.206 | 189.451 189.451 41.526 | 189.094 189.094 41.207 | 115.997 184.17 68.838 |
| α β γ | 90 90 120 | 90 90 120 | 90 90 120 | 90 90 90 |
| Unique reflections | 124813 (11420) | 109189 (10912) | 155226 (15252) | 67067 (6544) |
| Completeness (%) | 98.35 (90.13) | 99.72 (99.58) | 99.76 (97.99) | 99.73 (98.27) |
| Mean I/sigma(I) | 32.45 (2.51) | 43.53 (3.80) | 31.30 (2.57) | 16.61 (1.57) |
| Wilson B-factor | 14.97 | 12.1 | 12.34 | 42.78 |
| R-meas | 0.059 (0.384) | 0.070 (0.382) | 0.068 (0.521) | 0.121 (0.892) |
| CC1/2 | 0.995 (0.938) | 0.995 (0.848) | 1.00 (0.93) | 0.99 (0.719) |
| Data redundancy | 4.8 (3.9) | 4.5 (3.4) | 9.8 (7.9) | 6.5 (6.1) |
| **Refinement** |  |  |  |  |
| Reflections used in refinement | 124759 (11407) | 109056 (10909) | 155207 (15249) | 67054 (6544) |
| Reflections used for R-free | 6321 (556) | 5455 (563) | 7789 (786) | 3389 (337) |
| R-work | 0.1609 (0.2505) | 0.1508 (0.1863) | 0.1385 (0.1780) | 0.1702 (0.2391) |
| R-free | 0.1842 (0.2828) | 0.1719 (0.2085) | 0.1604 (0.2156) | 0.2088 (0.2947) |
| Number of non-hydrogen atoms | 5292 | 5516 | 5500 | 8956 |
| macromolecules | 4760 | 4687 | 4767 | 8292 |
| ligands | 65 | 165 | 158 | 214 |
| solvent | 467 | 664 | 575 | 450 |
| Protein residues | 597 | 601 | 607 | 1060 |
| RMS(bonds) | 0.016 | 0.019 | 0.017 | 0.009 |
| RMS(angles) | 1.49 | 1.79 | 1.63 | 1.03 |
| Ramachandran favored (%) | 97.96 | 97.61 | 97.79 | 97.03 |
| Ramachandran allowed (%) | 2.04 | 2.39 | 2.21 | 2.78 |
| Ramachandran outliers (%) | 0 | 0 | 0 | 0.19 |
| Rotamer outliers (%) | 0.19 | 0.58 | 0.38 | 0.22 |
| Clashscore | 4.57 | 3.08 | 5.54 | 5.08 |
| Average B-factor | 21.66 | 17.39 | 18.06 | 47.84 |
| macromolecules | 20.67 | 15.27 | 16.61 | 47.29 |
| ligands | 34.49 | 29.82 | 26.56 | 68.61 |
| solvent | 29.93 | 29.27 | 27.79 | 48.05 |

*Values in parentheses are for highest-resolution shell.
