# Supplementary material for: Molecular Insights into the Assembly and Functional Diversification of Typhoid Toxin
Source: mBio. 2022 Jan 11;13(1):e01916-21. doi: 10.1128/mbio.01916-21 (PMC8749428; doi:10.1128/mbio.01916-21)
Supplement: TABLE S2 [file mbio.01916-21-st002.docx]

**TABLE S2****A. PltC and PltB-binding to a customized sialoglycan microarray**

**PltC**

| **Glycan ID** | **Glycan Structures** | **Neu5Gc** | | **Neu5Ac** | |
| --- | --- | --- | --- | --- | --- |
|  |  | **ARFU^*^** | **SD^**^** | **ARFU** | **SD** |
| Gc/Ac-1 | Siaα2-3Galβ1-4GlcNAcβ1-2Manα1-6(Siaα2-3Galβ1-4GlcNAcβ1-2Manα1-3)Manβ1-4GlcNAcβ1-4GlcNAc- | 25036.17 | 2385.22 | 14209.67 | 1657.24 |
| Gc/Ac-2 | Siaα2-6Galβ1-4GlcNAcβ1-2Manα1-6(Siaα2-6Galβ1-4GlcNAcβ1-2Manα1-3)Manβ1-4GlcNAcβ1-4GlcNAc- | 26740.5 | 22672.47 | 55936.83 | 8649.76 |
| Gc/Ac-3 | Siaα2-3Galβ1-4(Fucα1-3)GlcNAcβ1-2Manα1-6(Siaα2-3Galβ1-4(Fucα1-3)GlcNAcβ1-2Manα1-3)Manβ1-4GlcNAcβ1-4GlcNAc- | 26994.5 | 8407.64 | 3490.33 | 3885.18 |
| Gc/Ac-4 | Manα1-6(Manα1-3)Manα1-6(Siaα2-3Galβ1-4GlcNAcβ1-2Manα1-3)Manβ1-4GlcNAcβ1-4GlcNAc- | 10644.33 | 12456.43 | 6511.67 | 1188.8 |
| Gc/Ac-5 | Manα1-6(Manα1-3)Manα1-6(Siaα2-6Galβ1-4GlcNAcβ1-2Manα1-3)Manβ1-4GlcNAcβ1-4GlcNAc- | 23969 | 963.75 | 13961.5 | 5376.14 |
| Gc/Ac-6 | Manα1-6(Manα1-3)Manα1-6(Siaα2-3Galβ1-4(Fucα1-3)GlcNAcβ1-2Manα1-3)Manβ1-4GlcNAcβ1-4GlcNAc- | 8570 | 4466.3 | -1492.33 | 724.67 |
| Gc/Ac-7 | Siaα2-3Galβ1-4GlcNAcβ1-2Manα1-3Manβ1-4GlcNAcβ1-4GlcNAc- | 14163.5 | 2866.59 | 30454.5 | 3991.31 |
| Gc/Ac-8 | Siaα2-6Galβ1-4GlcNAcβ1-2Manα1-3Manβ1-4GlcNAcβ1-4GlcNAc- | 30176.67 | 7056.78 | 33836.67 | 3630.69 |
| Gc/Ac-9 | Siaα2-3Galβ1-4(Fucα1-3)GlcNAcβ1-2Manα1-3Manβ1-4GlcNAcβ1-4GlcNAc- | 13146.17 | 11405.86 | 35157.17 | 1181.29 |
| Gc/Ac-10 | Manα1-6(Siaα2-3Galβ1-4GlcNAcβ1-2Manα1-3)Manβ1-4GlcNAcβ1-4GlcNAc- | 22177 | 1907.99 | 14876.83 | 3418.27 |
| Gc/Ac-11 | Manα1-6(Siaα2-6Galβ1-4GlcNAcβ1-2Manα1-3)Manβ1-4GlcNAcβ1-4GlcNAc- | 12357.17 | 5288.74 | -375.83 | 932.59 |
| Gc/Ac-12 | Siaα2-3Galβ1-4GlcNAcβ1-2Manα1-6Manβ1-4GlcNAcβ1-4GlcNAc- | 8246 | 3154.13 | 238.67 | 1963.75 |
| Gc/Ac-13 | Siaα2-6Galβ1-4GlcNAcβ1-2Manα1-6Manβ1-4GlcNAcβ1-4GlcNAc- | 20705.67 | 2592.06 | 3914.17 | 4594.13 |
| Gc/Ac-14 | Siaα2-3Galβ1-4(Fucα1-3)GlcNAcβ1-2Manα1-6Manβ1-4GlcNAcβ1-4GlcNAc- | 7229.5 | 6015.18 | 32345.33 | 1894.48 |
| Gc/Ac-15 | Siaα2-3Galβ1-4GlcNAcβ1-2Manα1-6(Manα1-3)Manβ1-4GlcNAcβ1-4GlcNAc- | 8271 | 2269.5 | 2998.5 | 192.1 |
| Gc/Ac-16 | Siaα2-6Galβ1-4GlcNAcβ1-2Manα1-6(Manα1-3)Manβ1-4GlcNAcβ1-4GlcNAc- | 11325.33 | 7538.68 | 18216.33 | 15837.97 |
| Gc/Ac-17 | Siaα2-3Galβ1-4(Fucα1-3)GlcNAcβ1-2Manα1-6(Manα1-3)Manβ1-4GlcNAcβ1-4GlcNAc- | -1832.33 | 1100.96 | 1536.17 | 631.88 |
| Gc/Ac-18 | GlcNAcβ1-2Manα1-6(Siaα2-3Galβ1-4GlcNAcβ1-2Manα1-3)Manβ1-4GlcNAcβ1-4GlcNAc- | 4217.5 | 4044.72 | 797.33 | 439.81 |
| Gc/Ac-19 | GlcNAcβ1-2Manα1-6(Siaα2-6Galβ1-4GlcNAcβ1-2Manα1-3)Manβ1-4GlcNAcβ1-4GlcNAc- | 16729.67 | 4479.34 | 1138.67 | 474.57 |
| Gc/Ac-20 | GlcNAcβ1-2Manα1-6(Siaα2-3Galβ1-4(Fucα1-3)GlcNAcβ1-2Manα1-3)Manβ1-4GlcNAcβ1-4GlcNAc- | 19365.33 | 11059.68 | 384.5 | 249.05 |
| Gc/Ac-21 | Galβ1-4GlcNAcβ1-2Manα1-6(Siaα2-3Galβ1-4GlcNAcβ1-2Manα1-3)Manβ1-4GlcNAcβ1-4GlcNAc- | -960.83 | 1427.16 | -1392 | 572.69 |
| Gc/Ac-22 | Galβ1-4GlcNAcβ1-2Manα1-6(Siaα2-6Galβ1-4GlcNAcβ1-2Manα1-3)Manβ1-4GlcNAcβ1-4GlcNAc- | -979.17 | 706.65 | 2.17 | 450.26 |
| Gc/Ac-23 | Galβ1-4GlcNAcβ1-2Manα1-6(Siaα2-3Galβ1-4(Fucα1-3)GlcNAcβ1-2Manα1-3)Manβ1-4GlcNAcβ1-4GlcNAc- | 5627 | 2756 | 492.33 | 763.83 |
| Gc/Ac-24 | Siaα2-3Galβ1-4GlcNAcβ1-2Manα1-6(Siaα2-6Galβ1-4GlcNAcβ1-2Manα1-3)Manβ1-4GlcNAcβ1-4GlcNAc- | 24652.33 | 4559.82 | 1989.5 | 921.82 |
| Gc/Ac-25 | Siaα2-3Galβ1-4GlcNAcβ1-2Manα1-6(Galβ1-4(Fucα1-3)GlcNAcβ1-2Manα1-3)Manβ1-4GlcNAcβ1-4GlcNAc- | 1700.33 | 752.4 | 684.17 | 246.79 |
| Gc/Ac-26 | Siaα2-3Galβ1-4GlcNAcβ1-2Manα1-6(Siaα2-3Galβ1-4(Fucα1-3)GlcNAcβ1-2Manα1-3)Manβ1-4GlcNAcβ1-4GlcNAc- | 20738.33 | 7870.46 | -720.83 | 404.96 |
| Gc/Ac-27 | Siaα2-6Galβ1-4GlcNAcβ1-2Manα1-6(Galβ1-4(Fucα1-3)GlcNAcβ1-2Manα1-3)Manβ1-4GlcNAcβ1-4GlcNAc- | 993.83 | 2331.36 | 1612.5 | 844.28 |
| Gc/Blank-28 | Neu5Gcα2-6Galβ1-4GlcNAcβ1-2Manα1-6(Neu5Gcα2-3Galβ1-4(Fucα1-3)GlcNAcβ1-2Manα1-3)Manβ1-4GlcNAcβ1-4GlcNAc- | 29868.83 | 3229.56 | 0 | 0 |
| Gc/Ac-29 | Galβ1-4(Fucα1-3)GlcNAcβ1-2Manα1-6(Siaα2-3Galβ1-4(Fucα1-3)GlcNAcβ1-2Manα1-3)Manβ1-4GlcNAcβ1-4GlcNAc- | 2930.17 | 3484.11 | 421.83 | 369.44 |
| Gc/Ac-30 | Siaα2-3Galβ1-4GlcNAcβ1-2Manα1-6(GlcNAcβ1-2Manα1-3)Manβ1-4GlcNAcβ1-4GlcNAc- | 1006.67 | 1249.05 | 689.83 | 393.86 |
| Gc/Ac-31 | Siaα2-6Galβ1-4GlcNAcβ1-2Manα1-6(GlcNAcβ1-2Manα1-3)Manβ1-4GlcNAcβ1-4GlcNAc- | -1391.5 | 446.44 | 986.17 | 829.19 |
| Gc/Ac-32 | Siaα2-3Galβ1-4(Fucα1-3)GlcNAcβ1-2Manα1-6(GlcNAcβ1-2Manα1-3)Manβ1-4GlcNAcβ1-4GlcNAc- | -1256.5 | 309.1 | -322.5 | 56.06 |
| Gc/Ac-33 | Siaα2-3Galβ1-4GlcNAcβ1-2Manα1-6(Galβ1-4GlcNAcβ1-2Manα1-3)Manβ1-4GlcNAcβ1-4GlcNAc- | 969.67 | 415.42 | 2224.67 | 447.54 |
| Gc/Ac-34 | Siaα2-6Galβ1-4GlcNAcβ1-2Manα1-6(Galβ1-4GlcNAcβ1-2Manα1-3)Manβ1-4GlcNAcβ1-4GlcNAc- | 3178.5 | 1261.35 | 6064 | 553.59 |
| Gc/Ac-35 | Siaα2-3Galβ1-4(Fucα1-3)GlcNAcβ1-2Manα1-6(Galβ1-4GlcNAcβ1-2Manα1-3)Manβ1-4GlcNAcβ1-4GlcNAc- | 1129.17 | 915.49 | 738.67 | 192.02 |
| Gc/Ac-36 | Siaα2-6Galβ1-4GlcNAcβ1-2Manα1-6(Siaα2-3Galβ1-4GlcNAcβ1-2Manα1-3)Manβ1-4GlcNAcβ1-4GlcNAc- | 4722.83 | 9283.34 | 61032.5 | 3135.63 |
| Gc/Blank-37 | Neu5Gcα2-3Galβ1-4(Fucα1-3)GlcNAcβ1-2Manα1-6(Neu5Gcα2-3Galβ1-4GlcNAcβ1-2Manα1-3)Manβ1-4GlcNAcβ1-4GlcNAc- | 2350.33 | 1813.29 | 0 | 0 |
| Gc/Blank-38 | Neu5Gcα2-3Galβ1-4(Fucα1-3)GlcNAcβ1-2Manα1-6(Neu5Gcα2-6Galβ1-4GlcNAcβ1-2Manα1-3)Manβ1-4GlcNAcβ1-4GlcNAc- | 16654 | 7740.22 | 0 | 0 |
| Gc/Ac-39 | Siaα2-3Galβ1-4(Fucα1-3)GlcNAcβ1-2Manα1-6(Galβ1-4(Fucα1-3)GlcNAcβ1-2Manα1-3)Manβ1-4GlcNAcβ1-4GlcNAc- | 1198.33 | 424.38 | 287.5 | 221.61 |
| Gc/Blank-40 | Neu5Acα2-6Galβ1-4GlcNAcβ1-2Manα1-6(Neu5Gcα2-6Galβ1-4GlcNAcβ1-2Manα1-3)Manβ1-4GlcNAcβ1-4GlcNAc- | 12768.33 | 2830.26 | 0 | 0 |
| Gc/Blank-41 | Neu5Gcα2-6Galβ1-4GlcNAcβ1-2Manα1-6(Neu5Acα2-6Galβ1-4GlcNAcβ1-2Manα1-3)Manβ1-4GlcNAcβ1-4GlcNAc- | 6715.5 | 1271.62 | 0 | 0 |

**PltB**

| **Glycan ID** | **Glycan Structures** | **Neu5Gc** | | **Neu5Ac** | |
| --- | --- | --- | --- | --- | --- |
|  |  | **ARFU^*^** | **SD^**^** | **ARFU** | **SD** |
| Gc/Ac-1 | Siaα2-3Galβ1-4GlcNAcβ1-2Manα1-6(Siaα2-3Galβ1-4GlcNAcβ1-2Manα1-3)Manβ1-4GlcNAcβ1-4GlcNAc- | -797 | 231.63 | 10724.5 | 744.31 |
| Gc/Ac-2 | Siaα2-6Galβ1-4GlcNAcβ1-2Manα1-6(Siaα2-6Galβ1-4GlcNAcβ1-2Manα1-3)Manβ1-4GlcNAcβ1-4GlcNAc- | -778.5 | 612.07 | 26704.16 | 1870.94 |
| Gc/Ac-3 | Siaα2-3Galβ1-4(Fucα1-3)GlcNAcβ1-2Manα1-6(Siaα2-3Galβ1-4(Fucα1-3)GlcNAcβ1-2Manα1-3)Manβ1-4GlcNAcβ1-4GlcNAc- | -1036 | 367.77 | 1146.33 | 808.84 |
| Gc/Ac-4 | Manα1-6(Manα1-3)Manα1-6(Siaα2-3Galβ1-4GlcNAcβ1-2Manα1-3)Manβ1-4GlcNAcβ1-4GlcNAc- | -1538 | 309.28 | 6446.16 | 46.01 |
| Gc/Ac-5 | Manα1-6(Manα1-3)Manα1-6(Siaα2-6Galβ1-4GlcNAcβ1-2Manα1-3)Manβ1-4GlcNAcβ1-4GlcNAc- | -721.17 | 276.43 | 3450.66 | 3577.53 |
| Gc/Ac-6 | Manα1-6(Manα1-3)Manα1-6(Siaα2-3Galβ1-4(Fucα1-3)GlcNAcβ1-2Manα1-3)Manβ1-4GlcNAcβ1-4GlcNAc- | -674.84 | 485.04 | 3276.83 | 930.8 |
| Gc/Ac-7 | Siaα2-3Galβ1-4GlcNAcβ1-2Manα1-3Manβ1-4GlcNAcβ1-4GlcNAc- | -489.5 | 554.15 | 19614.5 | 5685.13 |
| Gc/Ac-8 | Siaα2-6Galβ1-4GlcNAcβ1-2Manα1-3Manβ1-4GlcNAcβ1-4GlcNAc- | -631.67 | 549.83 | 12439.66 | 227.37 |
| Gc/Ac-9 | Siaα2-3Galβ1-4(Fucα1-3)GlcNAcβ1-2Manα1-3Manβ1-4GlcNAcβ1-4GlcNAc- | -503 | 405.87 | 12198 | 1995.1 |
| Gc/Ac-10 | Manα1-6(Siaα2-3Galβ1-4GlcNAcβ1-2Manα1-3)Manβ1-4GlcNAcβ1-4GlcNAc- | -752 | 236.41 | 12237.66 | 232.26 |
| Gc/Ac-11 | Manα1-6(Siaα2-6Galβ1-4GlcNAcβ1-2Manα1-3)Manβ1-4GlcNAcβ1-4GlcNAc- | -851.34 | 271.18 | 2561.66 | 1278.64 |
| Gc/Ac-12 | Siaα2-3Galβ1-4GlcNAcβ1-2Manα1-6Manβ1-4GlcNAcβ1-4GlcNAc- | -314 | 389.75 | 5489.83 | 1979.61 |
| Gc/Ac-13 | Siaα2-6Galβ1-4GlcNAcβ1-2Manα1-6Manβ1-4GlcNAcβ1-4GlcNAc- | -565.17 | 972.53 | 1730.5 | 1906.3 |
| Gc/Ac-14 | Siaα2-3Galβ1-4(Fucα1-3)GlcNAcβ1-2Manα1-6Manβ1-4GlcNAcβ1-4GlcNAc- | -1049.84 | 1006.59 | 23887.83 | 853.04 |
| Gc/Ac-15 | Siaα2-3Galβ1-4GlcNAcβ1-2Manα1-6(Manα1-3)Manβ1-4GlcNAcβ1-4GlcNAc- | -1170 | 128.12 | 1384.33 | 1343.28 |
| Gc/Ac-16 | Siaα2-6Galβ1-4GlcNAcβ1-2Manα1-6(Manα1-3)Manβ1-4GlcNAcβ1-4GlcNAc- | -586.34 | 412.37 | 4876.5 | 519.64 |
| Gc/Ac-17 | Siaα2-3Galβ1-4(Fucα1-3)GlcNAcβ1-2Manα1-6(Manα1-3)Manβ1-4GlcNAcβ1-4GlcNAc- | -134.84 | 781.47 | 4279.5 | 916.6 |
| Gc/Ac-18 | GlcNAcβ1-2Manα1-6(Siaα2-3Galβ1-4GlcNAcβ1-2Manα1-3)Manβ1-4GlcNAcβ1-4GlcNAc- | -682 | 465.91 | 1301.33 | 81.96 |
| Gc/Ac-19 | GlcNAcβ1-2Manα1-6(Siaα2-6Galβ1-4GlcNAcβ1-2Manα1-3)Manβ1-4GlcNAcβ1-4GlcNAc- | -576.34 | 633.28 | 1040 | 512.28 |
| Gc/Ac-20 | GlcNAcβ1-2Manα1-6(Siaα2-3Galβ1-4(Fucα1-3)GlcNAcβ1-2Manα1-3)Manβ1-4GlcNAcβ1-4GlcNAc- | -1433.34 | 366.11 | 480.5 | 830.33 |
| Gc/Ac-21 | Galβ1-4GlcNAcβ1-2Manα1-6(Siaα2-3Galβ1-4GlcNAcβ1-2Manα1-3)Manβ1-4GlcNAcβ1-4GlcNAc- | -62 | 1056.4 | 1520.5 | 454.5 |
| Gc/Ac-22 | Galβ1-4GlcNAcβ1-2Manα1-6(Siaα2-6Galβ1-4GlcNAcβ1-2Manα1-3)Manβ1-4GlcNAcβ1-4GlcNAc- | 832.33 | 578.65 | 1722.16 | 317.08 |
| Gc/Ac-23 | Galβ1-4GlcNAcβ1-2Manα1-6(Siaα2-3Galβ1-4(Fucα1-3)GlcNAcβ1-2Manα1-3)Manβ1-4GlcNAcβ1-4GlcNAc- | -378.17 | 526.6 | 1651.16 | 490.28 |
| Gc/Ac-24 | Siaα2-3Galβ1-4GlcNAcβ1-2Manα1-6(Siaα2-6Galβ1-4GlcNAcβ1-2Manα1-3)Manβ1-4GlcNAcβ1-4GlcNAc- | 494.5 | 337.28 | 2056.83 | 381.42 |
| Gc/Ac-25 | Siaα2-3Galβ1-4GlcNAcβ1-2Manα1-6(Galβ1-4(Fucα1-3)GlcNAcβ1-2Manα1-3)Manβ1-4GlcNAcβ1-4GlcNAc- | -1116.5 | 240.55 | 429.5 | 446.9 |
| Gc/Ac-26 | Siaα2-3Galβ1-4GlcNAcβ1-2Manα1-6(Siaα2-3Galβ1-4(Fucα1-3)GlcNAcβ1-2Manα1-3)Manβ1-4GlcNAcβ1-4GlcNAc- | -31.5 | 319.41 | 1649.16 | 325.09 |
| Gc/Ac-27 | Siaα2-6Galβ1-4GlcNAcβ1-2Manα1-6(Galβ1-4(Fucα1-3)GlcNAcβ1-2Manα1-3)Manβ1-4GlcNAcβ1-4GlcNAc- | -6.34 | 227.92 | 1744 | 321.52 |
| Gc/Blank-28 | Neu5Gcα2-6Galβ1-4GlcNAcβ1-2Manα1-6(Neu5Gcα2-3Galβ1-4(Fucα1-3)GlcNAcβ1-2Manα1-3)Manβ1-4GlcNAcβ1-4GlcNAc- | -309.84 | 588.29 | 0 | 0 |
| Gc/Ac-29 | Galβ1-4(Fucα1-3)GlcNAcβ1-2Manα1-6(Siaα2-3Galβ1-4(Fucα1-3)GlcNAcβ1-2Manα1-3)Manβ1-4GlcNAcβ1-4GlcNAc- | -488.5 | 454.96 | 1142.83 | 211.47 |
| Gc/Ac-30 | Siaα2-3Galβ1-4GlcNAcβ1-2Manα1-6(GlcNAcβ1-2Manα1-3)Manβ1-4GlcNAcβ1-4GlcNAc- | -1694.5 | 273.4 | 2209.5 | 615.06 |
| Gc/Ac-31 | Siaα2-6Galβ1-4GlcNAcβ1-2Manα1-6(GlcNAcβ1-2Manα1-3)Manβ1-4GlcNAcβ1-4GlcNAc- | 116.5 | 283.25 | 2570.83 | 42.3 |
| Gc/Ac-32 | Siaα2-3Galβ1-4(Fucα1-3)GlcNAcβ1-2Manα1-6(GlcNAcβ1-2Manα1-3)Manβ1-4GlcNAcβ1-4GlcNAc- | -285.5 | 475.59 | 1498.5 | 177.22 |
| Gc/Ac-33 | Siaα2-3Galβ1-4GlcNAcβ1-2Manα1-6(Galβ1-4GlcNAcβ1-2Manα1-3)Manβ1-4GlcNAcβ1-4GlcNAc- | 155.66 | 831.33 | 16182.5 | 858.81 |
| Gc/Ac-34 | Siaα2-6Galβ1-4GlcNAcβ1-2Manα1-6(Galβ1-4GlcNAcβ1-2Manα1-3)Manβ1-4GlcNAcβ1-4GlcNAc- | 153.83 | 811.9 | 8539.33 | 129.2 |
| Gc/Ac-35 | Siaα2-3Galβ1-4(Fucα1-3)GlcNAcβ1-2Manα1-6(Galβ1-4GlcNAcβ1-2Manα1-3)Manβ1-4GlcNAcβ1-4GlcNAc- | -855.34 | 48.05 | 4299.5 | 286.2 |
| Gc/Ac-36 | Siaα2-6Galβ1-4GlcNAcβ1-2Manα1-6(Siaα2-3Galβ1-4GlcNAcβ1-2Manα1-3)Manβ1-4GlcNAcβ1-4GlcNAc- | 249.5 | 101.76 | 24724.33 | 704.13 |
| Gc/Blank-37 | Neu5Gcα2-3Galβ1-4(Fucα1-3)GlcNAcβ1-2Manα1-6(Neu5Gcα2-3Galβ1-4GlcNAcβ1-2Manα1-3)Manβ1-4GlcNAcβ1-4GlcNAc- | 532.5 | 764.44 | 0 | 0 |
| Gc/Blank-38 | Neu5Gcα2-3Galβ1-4(Fucα1-3)GlcNAcβ1-2Manα1-6(Neu5Gcα2-6Galβ1-4GlcNAcβ1-2Manα1-3)Manβ1-4GlcNAcβ1-4GlcNAc- | 104 | 758.53 | 0 | 0 |
| Gc/Ac-39 | Siaα2-3Galβ1-4(Fucα1-3)GlcNAcβ1-2Manα1-6(Galβ1-4(Fucα1-3)GlcNAcβ1-2Manα1-3)Manβ1-4GlcNAcβ1-4GlcNAc- | 17.16 | 484.39 | 2370.33 | 247.9 |
| Gc/Blank-40 | Neu5Acα2-6Galβ1-4GlcNAcβ1-2Manα1-6(Neu5Gcα2-6Galβ1-4GlcNAcβ1-2Manα1-3)Manβ1-4GlcNAcβ1-4GlcNAc- | -275.5 | 351.33 | 0 | 0 |
| Gc/Blank-41 | Neu5Gcα2-6Galβ1-4GlcNAcβ1-2Manα1-6(Neu5Acα2-6Galβ1-4GlcNAcβ1-2Manα1-3)Manβ1-4GlcNAcβ1-4GlcNAc- | 1012.16 | 387.88 | 0 | 0 |

*, Average RFU - RFU of Negative Control

**, Standard deviation

**TABLE S2B.** **Analysis of fine ligand specificity of PltC and PltB**

**PltC**

| **Glycan ID** | **Glycan Structures** | **Neu5Gc** | | **Neu5Ac** | |
| --- | --- | --- | --- | --- | --- |
|  |  | **Mean** | **SD^*^** | **Mean** | **SD** |
| Gc/Ac-2 | Siaα2-6Galβ1-4GlcNAcβ1-2Manα1-6(Siaα2-6Galβ1-4GlcNAcβ1-2Manα1-3)Manβ1-4GlcNAcβ1-4GlcNAc- | 26740.5 | 22672.47 | 55936.83 | 8649.76 |
| Gc/Ac-8 | Siaα2-6Galβ1-4GlcNAcβ1-2Manα1-3Manβ1-4GlcNAcβ1-4GlcNAc- | 30176.67 | 7056.78 | 33836.67 | 3630.69 |
| Gc/Ac-13 | Siaα2-6Galβ1-4GlcNAcβ1-2Manα1-6Manβ1-4GlcNAcβ1-4GlcNAc- | 20705.67 | 2592.06 | 3914.17 | 4594.13 |
| Gc/Ac-1 | Siaα2-3Galβ1-4GlcNAcβ1-2Manα1-6(Siaα2-3Galβ1-4GlcNAcβ1-2Manα1-3)Manβ1-4GlcNAcβ1-4GlcNAc- | 25036.17 | 2385.22 | 14209.67 | 1657.24 |
| Gc/Ac-7 | Siaα2-3Galβ1-4GlcNAcβ1-2Manα1-3Manβ1-4GlcNAcβ1-4GlcNAc- | 14163.5 | 2866.59 | 30454.5 | 3991.31 |
| Gc/Ac-12 | Siaα2-3Galβ1-4GlcNAcβ1-2Manα1-6Manβ1-4GlcNAcβ1-4GlcNAc- | 8246 | 3154.13 | 238.67 | 1963.75 |
| Gc/Ac-26 | Siaα2-3Galβ1-4GlcNAcβ1-2Manα1-6(Siaα2-3Galβ1-4(Fucα1-3)GlcNAcβ1-2Manα1-3)Manβ1-4GlcNAcβ1-4GlcNAc- | 20738.33 | 7870.46 | -720.83 | 404.96 |
| Gc/Ac-9 | Siaα2-3Galβ1-4(Fucα1-3)GlcNAcβ1-2Manα1-3Manβ1-4GlcNAcβ1-4GlcNAc- | 13146.17 | 11405.86 | 35157.17 | 1181.29 |
| Gc/Ac-12 | Siaα2-3Galβ1-4GlcNAcβ1-2Manα1-6Manβ1-4GlcNAcβ1-4GlcNAc- | 8246 | 3154.13 | 238.67 | 1963.75 |
| Gc/Ac-3 | Siaα2-3Galβ1-4(Fucα1-3)GlcNAcβ1-2Manα1-6(Siaα2-3Galβ1-4(Fucα1-3)GlcNAcβ1-2Manα1-3)Manβ1-4GlcNAcβ1-4GlcNAc- | 26994.5 | 8407.64 | 3490.33 | 3885.18 |
| Gc/Ac-9 | Siaα2-3Galβ1-4(Fucα1-3)GlcNAcβ1-2Manα1-3Manβ1-4GlcNAcβ1-4GlcNAc- | 13146.17 | 11405.86 | 35157.17 | 1181.29 |
| Gc/Ac-14 | Siaα2-3Galβ1-4(Fucα1-3)GlcNAcβ1-2Manα1-6Manβ1-4GlcNAcβ1-4GlcNAc- | 7229.5 | 6015.18 | 32345.33 | 1894.48 |

**PltB**

| **Glycan ID** | **Glycan Structures** | **Neu5Ac** | |
| --- | --- | --- | --- |
|  |  | **Mean** | **SD** |
| Gc/Ac-2 | Siaα2-6Galβ1-4GlcNAcβ1-2Manα1-6(Siaα2-6Galβ1-4GlcNAcβ1-2Manα1-3)Manβ1-4GlcNAcβ1-4GlcNAc- | 26704.16 | 1870.94 |
| Gc/Ac-8 | Siaα2-6Galβ1-4GlcNAcβ1-2Manα1-3Manβ1-4GlcNAcβ1-4GlcNAc- | 12439.66 | 227.37 |
| Gc/Ac-13 | Siaα2-6Galβ1-4GlcNAcβ1-2Manα1-6Manβ1-4GlcNAcβ1-4GlcNAc- | 1730.5 | 1906.3 |
| Gc/Ac-1 | Siaα2-3Galβ1-4GlcNAcβ1-2Manα1-6(Siaα2-3Galβ1-4GlcNAcβ1-2Manα1-3)Manβ1-4GlcNAcβ1-4GlcNAc- | 10724.5 | 744.31 |
| Gc/Ac-7 | Siaα2-3Galβ1-4GlcNAcβ1-2Manα1-3Manβ1-4GlcNAcβ1-4GlcNAc- | 19614.5 | 5685.13 |
| Gc/Ac-12 | Siaα2-3Galβ1-4GlcNAcβ1-2Manα1-6Manβ1-4GlcNAcβ1-4GlcNAc- | 5489.83 | 1979.61 |
| Gc/Ac-26 | Siaα2-3Galβ1-4GlcNAcβ1-2Manα1-6(Siaα2-3Galβ1-4(Fucα1-3)GlcNAcβ1-2Manα1-3)Manβ1-4GlcNAcβ1-4GlcNAc- | 1649.16 | 325.09 |
| Gc/Ac-9 | Siaα2-3Galβ1-4(Fucα1-3)GlcNAcβ1-2Manα1-3Manβ1-4GlcNAcβ1-4GlcNAc- | 12198 | 1995.1 |
| Gc/Ac-12 | Siaα2-3Galβ1-4GlcNAcβ1-2Manα1-6Manβ1-4GlcNAcβ1-4GlcNAc- | 5489.83 | 1979.61 |
| Gc/Ac-3 | Siaα2-3Galβ1-4(Fucα1-3)GlcNAcβ1-2Manα1-6(Siaα2-3Galβ1-4(Fucα1-3)GlcNAcβ1-2Manα1-3)Manβ1-4GlcNAcβ1-4GlcNAc- | 1146.33 | 808.84 |
| Gc/Ac-9 | Siaα2-3Galβ1-4(Fucα1-3)GlcNAcβ1-2Manα1-3Manβ1-4GlcNAcβ1-4GlcNAc- | 12198 | 1995.1 |
| Gc/Ac-14 | Siaα2-3Galβ1-4(Fucα1-3)GlcNAcβ1-2Manα1-6Manβ1-4GlcNAcβ1-4GlcNAc- | 23887.83 | 853.04 |

*, Standard deviation

**TABLE S2C. Analysis of ligand specificity of PltC and PltB**

**PltC**

| **Glycan ID** | **Glycan Structures** | **Normalized Mean RFU** | |
| --- | --- | --- | --- |
|  |  | **Neu5Gc** | **Neu5Ac** |
| Gc/Ac-1 | Siaα2-3Galβ1-4GlcNAcβ1-2Manα1-6(Siaα2-3Galβ1-4GlcNAcβ1-2Manα1-3)Manβ1-4GlcNAcβ1-4GlcNAc- | 41.02105 | 23.28214 |
| Gc/Ac-2 | Siaα2-6Galβ1-4GlcNAcβ1-2Manα1-6(Siaα2-6Galβ1-4GlcNAcβ1-2Manα1-3)Manβ1-4GlcNAcβ1-4GlcNAc- | 43.81354 | 91.65089 |
| Gc/Ac-3 | Siaα2-3Galβ1-4(Fucα1-3)GlcNAcβ1-2Manα1-6(Siaα2-3Galβ1-4(Fucα1-3)GlcNAcβ1-2Manα1-3)Manβ1-4GlcNAcβ1-4GlcNAc- | 44.22971 | 5.718806 |
| Gc/Ac-4 | Manα1-6(Manα1-3)Manα1-6(Siaα2-3Galβ1-4GlcNAcβ1-2Manα1-3)Manβ1-4GlcNAcβ1-4GlcNAc- | 17.44043 | 10.66918 |
| Gc/Ac-5 | Manα1-6(Manα1-3)Manα1-6(Siaα2-6Galβ1-4GlcNAcβ1-2Manα1-3)Manβ1-4GlcNAcβ1-4GlcNAc- | 39.27252 | 22.87552 |
| Gc/Ac-6 | Manα1-6(Manα1-3)Manα1-6(Siaα2-3Galβ1-4(Fucα1-3)GlcNAcβ1-2Manα1-3)Manβ1-4GlcNAcβ1-4GlcNAc- | 14.0417 | -2.44514 |
| Gc/Ac-7 | Siaα2-3Galβ1-4GlcNAcβ1-2Manα1-3Manβ1-4GlcNAcβ1-4GlcNAc- | 23.20649 | 49.89882 |
| Gc/Ac-8 | Siaα2-6Galβ1-4GlcNAcβ1-2Manα1-3Manβ1-4GlcNAcβ1-4GlcNAc- | 49.44361 | 55.44041 |
| Gc/Ac-9 | Siaα2-3Galβ1-4(Fucα1-3)GlcNAcβ1-2Manα1-3Manβ1-4GlcNAcβ1-4GlcNAc- | 21.53962 | 57.60401 |
| Gc/Ac-10 | Manα1-6(Siaα2-3Galβ1-4GlcNAcβ1-2Manα1-3)Manβ1-4GlcNAcβ1-4GlcNAc- | 36.33638 | 24.37526 |
| Gc/Ac-11 | Manα1-6(Siaα2-6Galβ1-4GlcNAcβ1-2Manα1-3)Manβ1-4GlcNAcβ1-4GlcNAc- | 20.24687 | -0.61579 |
| Gc/Ac-12 | Siaα2-3Galβ1-4GlcNAcβ1-2Manα1-6Manβ1-4GlcNAcβ1-4GlcNAc- | 13.51083 | 0.391054 |
| Gc/Ac-13 | Siaα2-6Galβ1-4GlcNAcβ1-2Manα1-6Manβ1-4GlcNAcβ1-4GlcNAc- | 33.92565 | 6.413255 |
| Gc/Ac-14 | Siaα2-3Galβ1-4(Fucα1-3)GlcNAcβ1-2Manα1-6Manβ1-4GlcNAcβ1-4GlcNAc- | 11.84533 | 52.9969 |
| Gc/Ac-15 | Siaα2-3Galβ1-4GlcNAcβ1-2Manα1-6(Manα1-3)Manβ1-4GlcNAcβ1-4GlcNAc- | 13.5518 | 4.912956 |
| Gc/Ac-16 | Siaα2-6Galβ1-4GlcNAcβ1-2Manα1-6(Manα1-3)Manβ1-4GlcNAcβ1-4GlcNAc- | 18.55623 | 29.84693 |
| Gc/Ac-17 | Siaα2-3Galβ1-4(Fucα1-3)GlcNAcβ1-2Manα1-6(Manα1-3)Manβ1-4GlcNAcβ1-4GlcNAc- | -3.00222 | 2.51697 |
| Gc/Ac-18 | GlcNAcβ1-2Manα1-6(Siaα2-3Galβ1-4GlcNAcβ1-2Manα1-3)Manβ1-4GlcNAcβ1-4GlcNAc- | 6.910253 | 1.306402 |
| Gc/Ac-19 | GlcNAcβ1-2Manα1-6(Siaα2-6Galβ1-4GlcNAcβ1-2Manα1-3)Manβ1-4GlcNAcβ1-4GlcNAc- | 27.41108 | 1.865678 |
| Gc/Ac-20 | GlcNAcβ1-2Manα1-6(Siaα2-3Galβ1-4(Fucα1-3)GlcNAcβ1-2Manα1-3)Manβ1-4GlcNAcβ1-4GlcNAc- | 31.72954 | 0.629992 |
| Gc/Ac-21 | Galβ1-4GlcNAcβ1-2Manα1-6(Siaα2-3Galβ1-4GlcNAcβ1-2Manα1-3)Manβ1-4GlcNAcβ1-4GlcNAc- | -1.57429 | -2.28075 |
| Gc/Ac-22 | Galβ1-4GlcNAcβ1-2Manα1-6(Siaα2-6Galβ1-4GlcNAcβ1-2Manα1-3)Manβ1-4GlcNAcβ1-4GlcNAc- | -1.60434 | 0.003555 |
| Gc/Ac-23 | Galβ1-4GlcNAcβ1-2Manα1-6(Siaα2-3Galβ1-4(Fucα1-3)GlcNAcβ1-2Manα1-3)Manβ1-4GlcNAcβ1-4GlcNAc- | 9.219678 | 0.806669 |
| Gc/Ac-24 | Siaα2-3Galβ1-4GlcNAcβ1-2Manα1-6(Siaα2-6Galβ1-4GlcNAcβ1-2Manα1-3)Manβ1-4GlcNAcβ1-4GlcNAc- | 40.39214 | 3.259739 |
| Gc/Ac-25 | Siaα2-3Galβ1-4GlcNAcβ1-2Manα1-6(Galβ1-4(Fucα1-3)GlcNAcβ1-2Manα1-3)Manβ1-4GlcNAcβ1-4GlcNAc- | 2.785942 | 1.120993 |
| Gc/Ac-26 | Siaα2-3Galβ1-4GlcNAcβ1-2Manα1-6(Siaα2-3Galβ1-4(Fucα1-3)GlcNAcβ1-2Manα1-3)Manβ1-4GlcNAcβ1-4GlcNAc- | 33.97916 | -1.18106 |
| Gc/Ac-27 | Siaα2-6Galβ1-4GlcNAcβ1-2Manα1-6(Galβ1-4(Fucα1-3)GlcNAcβ1-2Manα1-3)Manβ1-4GlcNAcβ1-4GlcNAc- | 1.628362 | 2.642035 |
| Gc/Ac-29 | Galβ1-4(Fucα1-3)GlcNAcβ1-2Manα1-6(Siaα2-3Galβ1-4(Fucα1-3)GlcNAcβ1-2Manα1-3)Manβ1-4GlcNAcβ1-4GlcNAc- | 4.800999 | 0.691156 |
| Gc/Ac-30 | Siaα2-3Galβ1-4GlcNAcβ1-2Manα1-6(GlcNAcβ1-2Manα1-3)Manβ1-4GlcNAcβ1-4GlcNAc- | 1.6494 | 1.130267 |
| Gc/Ac-31 | Siaα2-6Galβ1-4GlcNAcβ1-2Manα1-6(GlcNAcβ1-2Manα1-3)Manβ1-4GlcNAcβ1-4GlcNAc- | -2.27993 | 1.615811 |
| Gc/Ac-32 | Siaα2-3Galβ1-4(Fucα1-3)GlcNAcβ1-2Manα1-6(GlcNAcβ1-2Manα1-3)Manβ1-4GlcNAcβ1-4GlcNAc- | -2.05874 | -0.52841 |
| Gc/Ac-33 | Siaα2-3Galβ1-4GlcNAcβ1-2Manα1-6(Galβ1-4GlcNAcβ1-2Manα1-3)Manβ1-4GlcNAcβ1-4GlcNAc- | 1.588776 | 3.645058 |
| Gc/Ac-34 | Siaα2-6Galβ1-4GlcNAcβ1-2Manα1-6(Galβ1-4GlcNAcβ1-2Manα1-3)Manβ1-4GlcNAcβ1-4GlcNAc- | 5.207881 | 9.93569 |
| Gc/Ac-35 | Siaα2-3Galβ1-4(Fucα1-3)GlcNAcβ1-2Manα1-6(Galβ1-4GlcNAcβ1-2Manα1-3)Manβ1-4GlcNAcβ1-4GlcNAc- | 1.850113 | 1.21029 |
| Gc/Ac-36 | Siaα2-6Galβ1-4GlcNAcβ1-2Manα1-6(Siaα2-3Galβ1-4GlcNAcβ1-2Manα1-3)Manβ1-4GlcNAcβ1-4GlcNAc- | 7.738221 | 100 |
| Gc/Ac-39 | Siaα2-3Galβ1-4(Fucα1-3)GlcNAcβ1-2Manα1-6(Galβ1-4(Fucα1-3)GlcNAcβ1-2Manα1-3)Manβ1-4GlcNAcβ1-4GlcNAc- | 1.963429 | 0.471061 |

**PltB**

| **Glycan ID** | **Glycan Structures** | **Normalized Mean RFU** | |
| --- | --- | --- | --- |
|  |  | **Neu5Gc** | **Neu5Ac** |
| Gc/Ac-1 | Siaα2-3Galβ1-4GlcNAcβ1-2Manα1-6(Siaα2-3Galβ1-4GlcNAcβ1-2Manα1-3)Manβ1-4GlcNAcβ1-4GlcNAc- | -2.98455 | 40.16041 |
| Gc/Ac-2 | Siaα2-6Galβ1-4GlcNAcβ1-2Manα1-6(Siaα2-6Galβ1-4GlcNAcβ1-2Manα1-3)Manβ1-4GlcNAcβ1-4GlcNAc- | -2.91528 | 100 |
| Gc/Ac-3 | Siaα2-3Galβ1-4(Fucα1-3)GlcNAcβ1-2Manα1-6(Siaα2-3Galβ1-4(Fucα1-3)GlcNAcβ1-2Manα1-3)Manβ1-4GlcNAcβ1-4GlcNAc- | -3.87955 | 4.292702 |
| Gc/Ac-4 | Manα1-6(Manα1-3)Manα1-6(Siaα2-3Galβ1-4GlcNAcβ1-2Manα1-3)Manβ1-4GlcNAcβ1-4GlcNAc- | -5.7594 | 24.13916 |
| Gc/Ac-5 | Manα1-6(Manα1-3)Manα1-6(Siaα2-6Galβ1-4GlcNAcβ1-2Manα1-3)Manβ1-4GlcNAcβ1-4GlcNAc- | -2.70059 | 12.92181 |
| Gc/Ac-6 | Manα1-6(Manα1-3)Manα1-6(Siaα2-3Galβ1-4(Fucα1-3)GlcNAcβ1-2Manα1-3)Manβ1-4GlcNAcβ1-4GlcNAc- | -2.5271 | 12.27086 |
| Gc/Ac-7 | Siaα2-3Galβ1-4GlcNAcβ1-2Manα1-3Manβ1-4GlcNAcβ1-4GlcNAc- | -1.83305 | 73.4511 |
| Gc/Ac-8 | Siaα2-6Galβ1-4GlcNAcβ1-2Manα1-3Manβ1-4GlcNAcβ1-4GlcNAc- | -2.36544 | 46.58323 |
| Gc/Ac-9 | Siaα2-3Galβ1-4(Fucα1-3)GlcNAcβ1-2Manα1-3Manβ1-4GlcNAcβ1-4GlcNAc- | -1.8836 | 45.67828 |
| Gc/Ac-10 | Manα1-6(Siaα2-3Galβ1-4GlcNAcβ1-2Manα1-3)Manβ1-4GlcNAcβ1-4GlcNAc- | -2.81604 | 45.82679 |
| Gc/Ac-11 | Manα1-6(Siaα2-6Galβ1-4GlcNAcβ1-2Manα1-3)Manβ1-4GlcNAcβ1-4GlcNAc- | -3.18804 | 9.592738 |
| Gc/Ac-12 | Siaα2-3Galβ1-4GlcNAcβ1-2Manα1-6Manβ1-4GlcNAcβ1-4GlcNAc- | -1.17585 | 20.55796 |
| Gc/Ac-13 | Siaα2-6Galβ1-4GlcNAcβ1-2Manα1-6Manβ1-4GlcNAcβ1-4GlcNAc- | -2.11641 | 6.480264 |
| Gc/Ac-14 | Siaα2-3Galβ1-4(Fucα1-3)GlcNAcβ1-2Manα1-6Manβ1-4GlcNAcβ1-4GlcNAc- | -3.93137 | 89.45359 |
| Gc/Ac-15 | Siaα2-3Galβ1-4GlcNAcβ1-2Manα1-6(Manα1-3)Manβ1-4GlcNAcβ1-4GlcNAc- | -4.38134 | 5.183949 |
| Gc/Ac-16 | Siaα2-6Galβ1-4GlcNAcβ1-2Manα1-6(Manα1-3)Manβ1-4GlcNAcβ1-4GlcNAc- | -2.19569 | 18.2612 |
| Gc/Ac-17 | Siaα2-3Galβ1-4(Fucα1-3)GlcNAcβ1-2Manα1-6(Manα1-3)Manβ1-4GlcNAcβ1-4GlcNAc- | -0.50494 | 16.02559 |
| Gc/Ac-18 | GlcNAcβ1-2Manα1-6(Siaα2-3Galβ1-4GlcNAcβ1-2Manα1-3)Manβ1-4GlcNAcβ1-4GlcNAc- | -2.55391 | 4.873136 |
| Gc/Ac-19 | GlcNAcβ1-2Manα1-6(Siaα2-6Galβ1-4GlcNAcβ1-2Manα1-3)Manβ1-4GlcNAcβ1-4GlcNAc- | -2.15824 | 3.894524 |
| Gc/Ac-20 | GlcNAcβ1-2Manα1-6(Siaα2-3Galβ1-4(Fucα1-3)GlcNAcβ1-2Manα1-3)Manβ1-4GlcNAcβ1-4GlcNAc- | -5.36748 | 1.799345 |
| Gc/Ac-21 | Galβ1-4GlcNAcβ1-2Manα1-6(Siaα2-3Galβ1-4GlcNAcβ1-2Manα1-3)Manβ1-4GlcNAcβ1-4GlcNAc- | -0.23217 | 5.693869 |
| Gc/Ac-22 | Galβ1-4GlcNAcβ1-2Manα1-6(Siaα2-6Galβ1-4GlcNAcβ1-2Manα1-3)Manβ1-4GlcNAcβ1-4GlcNAc- | 3.116855 | 6.449033 |
| Gc/Ac-23 | Galβ1-4GlcNAcβ1-2Manα1-6(Siaα2-3Galβ1-4(Fucα1-3)GlcNAcβ1-2Manα1-3)Manβ1-4GlcNAcβ1-4GlcNAc- | -1.41615 | 6.183156 |
| Gc/Ac-24 | Siaα2-3Galβ1-4GlcNAcβ1-2Manα1-6(Siaα2-6Galβ1-4GlcNAcβ1-2Manα1-3)Manβ1-4GlcNAcβ1-4GlcNAc- | 1.851771 | 7.702283 |
| Gc/Ac-25 | Siaα2-3Galβ1-4GlcNAcβ1-2Manα1-6(Galβ1-4(Fucα1-3)GlcNAcβ1-2Manα1-3)Manβ1-4GlcNAcβ1-4GlcNAc- | -4.181 | 1.608364 |
| Gc/Ac-26 | Siaα2-3Galβ1-4GlcNAcβ1-2Manα1-6(Siaα2-3Galβ1-4(Fucα1-3)GlcNAcβ1-2Manα1-3)Manβ1-4GlcNAcβ1-4GlcNAc- | -0.11796 | 6.175667 |
| Gc/Ac-27 | Siaα2-6Galβ1-4GlcNAcβ1-2Manα1-6(Galβ1-4(Fucα1-3)GlcNAcβ1-2Manα1-3)Manβ1-4GlcNAcβ1-4GlcNAc- | -0.02374 | 6.530818 |
| Gc/Ac-29 | Galβ1-4(Fucα1-3)GlcNAcβ1-2Manα1-6(Siaα2-3Galβ1-4(Fucα1-3)GlcNAcβ1-2Manα1-3)Manβ1-4GlcNAcβ1-4GlcNAc- | -1.8293 | 4.279595 |
| Gc/Ac-30 | Siaα2-3Galβ1-4GlcNAcβ1-2Manα1-6(GlcNAcβ1-2Manα1-3)Manβ1-4GlcNAcβ1-4GlcNAc- | -6.34545 | 8.273992 |
| Gc/Ac-31 | Siaα2-6Galβ1-4GlcNAcβ1-2Manα1-6(GlcNAcβ1-2Manα1-3)Manβ1-4GlcNAcβ1-4GlcNAc- | 0.436262 | 9.627077 |
| Gc/Ac-32 | Siaα2-3Galβ1-4(Fucα1-3)GlcNAcβ1-2Manα1-6(GlcNAcβ1-2Manα1-3)Manβ1-4GlcNAcβ1-4GlcNAc- | -1.06912 | 5.611485 |
| Gc/Ac-33 | Siaα2-3Galβ1-4GlcNAcβ1-2Manα1-6(Galβ1-4GlcNAcβ1-2Manα1-3)Manβ1-4GlcNAcβ1-4GlcNAc- | 0.582905 | 60.59917 |
| Gc/Ac-34 | Siaα2-6Galβ1-4GlcNAcβ1-2Manα1-6(Galβ1-4GlcNAcβ1-2Manα1-3)Manβ1-4GlcNAcβ1-4GlcNAc- | 0.576053 | 31.97753 |
| Gc/Ac-35 | Siaα2-3Galβ1-4(Fucα1-3)GlcNAcβ1-2Manα1-6(Galβ1-4GlcNAcβ1-2Manα1-3)Manβ1-4GlcNAcβ1-4GlcNAc- | -3.20302 | 16.10049 |
| Gc/Ac-36 | Siaα2-6Galβ1-4GlcNAcβ1-2Manα1-6(Siaα2-3Galβ1-4GlcNAcβ1-2Manα1-3)Manβ1-4GlcNAcβ1-4GlcNAc- | 0.934311 | 92.58606 |
| Gc/Ac-39 | Siaα2-3Galβ1-4(Fucα1-3)GlcNAcβ1-2Manα1-6(Galβ1-4(Fucα1-3)GlcNAcβ1-2Manα1-3)Manβ1-4GlcNAcβ1-4GlcNAc- | 0.06426 | 8.876257 |
